# Effect of a multifaceted intervention to improve clinical quality of care through stepwise certification (SafeCare) in health-care facilities in Tanzania: a cluster-randomised controlled trial

**DOI:** 10.1016/S2214-109X(21)00228-X

**Published:** 2021-08-04

**Authors:** Jessica J C King, Timothy Powell-Jackson, Christina Makungu, Nicole Spieker, Peter Risha, Abdallah Mkopi, Catherine Goodman

**Affiliations:** a London School of Hygiene & Tropical Medicine, London, UK; b Ifakara Health Institute, Dar es Salaam, Tanzania; c PharmAccess International, Amsterdam, Netherlands; d PharmAccess Tanzania, Dar es Salaam, Tanzania

## Abstract

**Background:**

Quality of care is consistently shown to be inadequate in health-care settings in many low-income and middle-income countries, including in private facilities, which are rapidly growing in number but often do not have effective quality stewardship mechanisms. The SafeCare programme aims to address this gap in quality of care, using a standards-based approach adapted to low-resource settings, involving assessments, mentoring, training, and access to loans, to improve clinical quality and facility business performance. We assessed the effect of the SafeCare programme on quality of patient care in faith-based and private for-profit facilities in Tanzania.

**Methods:**

In this cluster-randomised controlled trial, health facilities were eligible if they were dispensaries, health centres, or hospitals in the faith-based or private for-profit sectors in Tanzania. We randomly assigned facilities (1:1) using computer-generated stratified randomisation to receive the full SafeCare package (intervention) or an assessment only (control). Implementing staff and participants were masked to outcome measurement and the primary outcomes were measured by fieldworkers who had no knowledge of the study group allocation. The primary outcomes were health worker compliance with infection prevention and control (IPC) practices as measured by observation of provider–patient interactions, and correct case management of undercover standardised patients at endline (after a minimum of 18 months). Analyses were by modified intention to treat. The trial is registered with ISRCTN, ISRCTN93644888.

**Findings:**

Between March 7 and Nov 30, 2016, we enrolled and randomly assigned 237 health facilities to the intervention (n=118) or control (n=119). Nine facilities (seven intervention facilities and two control facilities) closed during the trial and were not included in the analysis. We observed 29 608 IPC indications in 5425 provider–patient interactions between Feb 7 and April 5, 2018. Health facilities received visits from 909 standardised patients between May 3 and June 12, 2018. Intervention facilities had a 4·4 percentage point (95% CI 0·9–7·7; p=0.015) higher mean SafeCare standards assessment score at endline than control facilities. However, there was no evidence of a difference in clinical quality between intervention and control groups at endline. Compliance with IPC practices was observed in 8181 (56·9%) of 14 366 indications in intervention facilities and 8336 (54·7%) of 15 242 indications in control facilities (absolute difference 2·2 percentage points, 95% CI −0·2 to −4·7; p=0·071). Correct management occurred in 120 (27·0%) of 444 standardised patients in the intervention group and in 136 (29·2%) of 465 in the control group (absolute difference −2·8 percentage points, 95% CI −8·6 to −3·1; p=0·36).

**Interpretation:**

SafeCare did not improve clinical quality as assessed by compliance with IPC practices and correct case management. The absence of effect on clinical quality could reflect a combination of insufficient intervention intensity, insufficient links between structural quality and care processes, scarcity of resources for quality improvement, and inadequate financial and regulatory incentives for improvement.

**Funding:**

UK Health Systems Research Initiative (Medical Research Council, Economic and Social Research Council, UK Department for International Development, Global Challenges Research Fund, and Wellcome Trust).

## Introduction

Quality of care has risen high up the global health policy agenda. Together with expanding coverage and financial protection, quality of care is a key component of health reforms inspired by universal health coverage.^1^, ^2^ Poor quality of care is estimated to result in approximately 5·7–8·4 million deaths per year in low-income and middle-income countries (LMICs),^3^ where patient care is consistently shown to be inadequate. In primary care in LMICs it is common for outpatients to receive less than half of the recommended clinical actions,^2^, ^4^ diagnoses for serious conditions are frequently incorrect,^5^, ^6^ and medicines are widely under-provided and over-provided.^7^ Health-care-associated infections continue to be an important threat to patient safety, reflecting poor infection prevention and control (IPC) practices.^2^, ^8^

**Research in context**

**Evidence before this study**

We searched for studies English, published up to December, 2019, that evaluated the effect of interventions working with private facilities on clinical quality of care in low-income and middle-income countries (LMICs), using PubMed, Web of Science, and Google Scholar (formal literature) and websites of key donor agencies, research institutions, private sector-focused consultancies, non-governmental organisations, and academic organisations including Private Health in Developing Countries, The World Bank, US Agency for International Development, Population Services International, Private Sector Partnerships One, Health Systems 20/20, Reproductive Health Vouchers, and the UK Department for International Development (grey literature), using terms related to the domains of “private”, “LMIC”, and a range of interventions (eg, “social franchising”). No evaluations of SafeCare or similar models in private facilities were identified. We reviewed controlled evaluations of accreditation and social franchising interventions, which have some similar components to SafeCare: three randomised controlled trials found no or weak efficacy with regard to clinical quality, while three non-randomised studies had mixed effects, and might have been affected by selection bias.

**Added value of this study**

This study is the first evaluation of the effect of SafeCare on clinical quality of care, and one of very few randomised controlled trials on any intervention aiming to improve quality in the private sector in LMICs. The SafeCare intervention led to a small improvement in adherence to SafeCare standards, but there was no effect on clinical quality of care.

**Implications of all the available evidence**

The SafeCare programme can improve structural quality, but there is no evidence that, at the intensity usually implemented, it has an effect on clinical quality of care. Improving clinical quality of care remains an important challenge and the evidence base does not yet provide guidance on which approaches are most promising.

There are concerns regarding quality of care in both the public and private health-care sector.^2^ The private sector is a substantial and growing provider of health services in many LMICs, responsible for around 63–67% of health care for sick children and 30–39% of maternal health care when averaged across 70 LMICs.^9^ Such high use of private health care partly reflects the established use of private medicine retailers and the provision of private health care by faith-based organisation facilities,^10^ but the use of for-profit private clinics and hospitals has grown rapidly in recent decades, reflecting urbanisation, the growth of the middle class, rising expectations of quality not met by the public sector, and empanelment of private facilities within social health insurance systems.^11^

Quality of care is extremely variable across such private health-care facilities,^12^, ^13^ and there are concerns about the scarcity of effective quality stewardship mechanisms for this sector.^14^ Statutory regulation of private facilities is typically very weak, with rare inspection and erratic enforcement, reflecting inadequate resources and capacity at the national level.^15^ Although health-care accreditation systems can, in theory, complement or substitute regulation of the facilities to some degree, the standards required by international accreditation bodies seem unattainable and the process too expensive for the vast majority of private facilities in LMICs. Alternative strategies to improve the quality of care in private facilities have been tried, including provider training, social franchising, and quality improvement cycles.^15^ However, the evidence base on the effectiveness of these strategies is scarce. Although there is some evidence that social franchising improves quality as perceived by patients, there is no robust evidence that any of these strategies improve clinical quality of care in an operational setting.^16^

These quality-of-care concerns led to the development of the SafeCare model, an innovative approach that adapts international accreditation standards to low-resource settings and supports health facilities to attain higher standards, recognising improvement through stepwise formal certification.^17^ SafeCare is a multifaceted intervention. At its heart are the SafeCare standards, a comprehensive set of measurable indicators, which are used to assess health facilities and assign them to one of five quality levels from the lowest (1) to the highest (5). The standards cover clinical care, ancillary services, and management processes, and are accredited by the International Society for Quality in Health Care, having been designed in partnership with the Joint Commission International and the Council for Health Service Accreditation of Southern Africa.^18^ SafeCare standards address the full range of a facility's operations, in contrast to many other quality improvement interventions that focus on specific clinical areas or target individual clinicians.^19^ Following a SafeCare assessment, facilities receive a tailored quality improvement plan, with implementation supported through clinical and business training, mentoring visits, and access to loans. Facilities receive a repeat SafeCare assessment after 1–2 years, with the intention that they gradually progress through the quality levels. With multiple components to the intervention there are numerous ways to improve, but the essence of the theory of change is that a greater adherence to SafeCare standards will lead to improvements in clinical quality, and business support will improve the facility's financial performance. The intention is to create a virtuous circle; improvements in quality attract more revenue from patients and institutional purchasers, and improved business performance facilitates greater investment in quality improvement. Launched in 2011, and currently the only programme of its kind, SafeCare has been implemented in 14 countries in sub-Saharan Africa, in over 2500 facilities, which receive over 5 million visits per month.

The objective of this study was to evaluate the effectiveness of SafeCare in improving clinical quality of care at the facility level. We chose to evaluate SafeCare in Tanzania, where it was being implemented on a large scale in private facilities across the country and with UK Government funding through the Human Development Innovation Fund. The private sector accounts for 29% of health facilities in Tanzania (15% faith-based and 14% for-profit), including hospitals and primary level facilities (health centres and smaller dispensaries).^20^ Historically, public oversight of private health facilities has been weak, although more recently there has been a strong commitment to enhancing quality in both the public and private sectors,^21^ since the roll-out of the government star rating initiative in 2015, which involves the inspection of all facilities and threats of closure for those that do not improve.^22^

## Methods

### Study design

We did a cluster-randomised controlled trial in private health facilities (clusters) in mainland Tanzania. Facilities were screened and recruited by implementing partners of PharmAccess (a non-profit organisation enabling access to better health care for people in sub-Saharan Africa) in Tanzania: the Association of Private Health Facilities in Tanzania (APHFTA), which represents mainly for-profit facilities, and the Christian Social Services Commission (CSSC), which represents mostly faith-based facilities. Facilities were recruited from the Northern, Eastern, Central, Southern, and Southern Highlands zones of Tanzania (Lake zone was excluded as SafeCare was already rolled out there before study commencement).

The study protocol was approved by the ethics committees of the Ifakara Health Institute (04–2016; Dar Es Salaam, Tanzania), the National Institute of Medical Research (IX/2415; Dar Es Salaam, Tanzania), and the London School of Hygiene & Tropical Medicine (10493; London, UK). Alongside this effectiveness study, we also did a process evaluation to understand implementation, mechanisms of effect, and context,^23^ which we draw on in the Discussion.

### Participants

Eligible facilities were dispensaries and health centres that were members of APHFTA, and dispensaries, health centres, and hospitals that were members of CSSC. Facilities were ineligible if they refused to provide consent to participate, provided specific services only (eg, mental health or maternity services), were tertiary hospitals, or had previously been exposed to SafeCare. We worked with APHFTA and CSSC to select a non-random list of 280 potentially eligible facilities for participation in the study (appendix p 2). The partner organisations approached these facilities to confirm their eligibility, explain the study, and obtain written informed consent from the facility manager. Study facilities were widely dispersed across both urban and rural areas, in 18 regions of mainland Tanzania.

Health workers participating in the study comprised those interviewed for a facility survey, those whose IPC practices were observed, and those visited by standardised patients. For the facility survey and IPC observations, written informed consent was obtained from the health worker before the start of data collection. At the time of the facility survey, facility managers were also asked to consent to visits from undercover standardised patients at an unspecified date within the next 3 months. Patients participating in the study were those directly observed during patient–provider interactions, and those who gave an exit interview before leaving the facility. For the observations, all patients were eligible if they or their adult caretaker gave verbal informed consent. Individuals aged younger than 18 years were excluded if they were not accompanied by an adult caretaker. For the exit interviews, patients were eligible if they were aged at least 18 years or were accompanied by an adult caretaker, had received curative outpatient care (therefore excluding routine visits for growth checks, immunisations, or antenatal care), and had completed their visit to the facility (including collecting prescribed treatments and making payments). Written informed consent was obtained from the patient or caretaker before the start of the interview.

### Randomisation and masking

We randomised facilities (clusters) to the SafeCare package (intervention) or an initial SafeCare assessment with no further support (control) in a ratio of 1:1. Randomisation was stratified by partner organisation, recruitment cohort, hospital or non-hospital, and geographical zone. Specifically, we randomly allocated health facilities to intervention or control, stratifying the sample so that the proportion allocated to each of the two groups was the same within each stratum. Randomisation of facilities was done using a computer-generated random number in Stata version 14.1.

A letter revealing the study group assignment was sealed in an envelope labelled with the facility name and given to a SafeCare quality assessor who opened the envelope to inform the facility manager of the allocation once written informed consent was given and the baseline SafeCare assessment was complete. PharmAccess staff, partner organisations, and facility participants were masked to study group allocation during recruitment and baseline assessment, but not for the rest of the study. They were also masked to outcome measurement—ie, they did not know what clinical quality metrics they were to be assessed on.

### Procedures

The SafeCare programme supports private health facilities to improve quality of care and business performance. The programme also includes quality-related policy support and advocacy at the health system level, but the trial intervention evaluated only the direct support to facilities. The SafeCare intervention was implemented by partners APHFTA and CSSC. Following initial sensitisation activities, SafeCare assessments were done by the implementation staff in both intervention and control facilities at baseline and, after a minimum of 18 months, at endline. Control facilities received a simple report of their assessment, but no further support. Separate data collection was done by the research team at endline, including observations of IPC practices, standardised patient visits, a survey of facility managers, and patient exit interviews (appendix p 3).

The initial SafeCare assessment assessed 170 standards grouped into 13 service elements: governance and management, human resource management, patient rights and access to care, management of information, risk management, primary health care, inpatient care, surgery and anaesthesia, laboratory services, diagnostic imaging, medication management, facility management services, and support services. Quality assessors measured how well facilities met the criteria, awarding an overall score between 0 and 100, and a performance level of one to five, with higher scores indicating better results. Quality assessors were employees of PharmAccess and were typically clinicians (clinical officers or nurses) who had completed a 70 h training programme with PharmAccess.

Facilities in the intervention group received a quality improvement plan that highlighted specific areas for improvement, actions to be taken, and the facility staff member responsible; mentoring visits from quality assessors to monitor progress on implementation of the quality improvement plan; training on topics such as infection control, waste management, customer care, business management, record keeping, and patient rights; and the opportunity to apply to the Medical Credit Fund, which is also part of the PharmAccess Group, for underwritten loans to fund specific quality improvement activities.

Quality assessors provided direct support to the managers of facilities in the intervention group, in the form of in-person mentoring visits to facilities, onsite training sessions, and offsite classroom training sessions for groups of facilities, attended by managers and clinicians. A full-time business analyst, employed by the Medical Credit Fund, made facilities aware of the SafeCare service during regional meetings attended by managers, and supported the writing of business cases and loan applications. Mentoring visits were intended to be quarterly (at least five visits were expected to take place in the 18–24-month period between baseline and endline SafeCare assessments), and staff from each facility were expected to attend at least two training sessions (either onsite or in the classroom).

We measured health provider compliance with IPC practices using observations of provider–patient interactions. 6 h of observation were done in each facility over the course of 1 day: 3 h in the consultation rooms, 1·5 h in the laboratory, and 1·5 h in the injection or dressing room. Fieldworkers recorded every indication (situation in which an infection risk to either patient, provider, or both was presented) and action (taken to mitigate the risk) using a tool that we developed according to WHO guidelines and previous studies^8^, ^24^, ^25^ and adapted to Tanzanian standards.^26^ The data were double entered on tablets using ODK Collect version 1.12.1. Health providers and patients were asked for written consent to participate in the study before observation commenced, but they were not informed of the focus on IPC practices or shown the tool. On the same day as observation, a survey of the health facility was done with the facility manager or representative, during which facility characteristics, patient numbers, and facility revenue were recorded. Also, up to eight patients per facility were given an exit interview. Implementation staff and study fieldworkers were not present at the same facility simultaneously.

Standardised patients were undercover healthy fieldworkers, trained to present at health facilities reporting specific symptoms and medical history, and to record the care they received. We describe the methods and the protocol for standardised patient safety in more detail in the appendix (pp 4–24). Briefly, on the basis of predefined selection criteria and a systematic review of the literature, we developed four standardised patient cases: asthma, non-malarial febrile illness, tuberculosis, and upper respiratory tract infection. Case descriptions and their corresponding correct management definitions are given in the appendix (p 4). Standardised patients were trained for 2 weeks, with extensive piloting and testing to ensure faithful reproduction of cases and recording of data. Each facility received each of the four standardised patient cases once, within the 3 months following consent being given. Standardised patients completed a debriefing questionnaire on a smartphone using ODK Collect version 1.12.1 immediately after the visit, and supervisors verified the information with the standardised patient the same day. A follow-up telephone survey was done with facility managers to assess whether any standardised patients were detected (appendix pp 21–22).

In terms of contamination, no control facilities received a quality improvement plan or mentoring visits, and seven control facilities attended some training. In practice, both intervention and control facilities had the opportunity to apply for a Medical Credit Fund loan, and two control facilities obtained such a loan during the study period.

### Outcomes

The two prespecified primary outcomes of the trial were observed compliance of health workers with IPC practices and correct case management of standardised patients at 18–24 months. The choice of outcomes reflected close consultation with staff involved in SafeCare's design and implementation, to capture the programme's broad aim of improving clinical quality of care, together with its emphasis on IPC practices.

Compliance with IPC practices was defined as the proportion of indications for which a correct action was done.^8^ Each of the 21 potential indications had a corresponding IPC action (appendix pp 29–30). For example, for each patient having blood drawn (indication), a new needle was required (action). Indications were coded as either compliant or non-compliant (binary).

Correct case management was defined as the proportion of standardised patients who were managed in accordance with the national standard treatment guidelines^27^ (appendix p 4). The definition of correct management was case-specific and it was coded as a binary outcome (correct or incorrect).

Secondary outcomes were the facility SafeCare assessment score (based on adherence to the 170 criteria, as described previously); patient experience-of-care score (based on responses to 21 Likert questions in the patient exit interview); patient out-of-pocket spending (including any consultation fee, and expenditure on medications and laboratory tests as measured by the standardised patients); facility caseload per month (measured by the facility survey using facility records from each medical department for the 3 preceding months); and facility revenue per month (measured by the facility survey using facility records on each income source for the 3 preceding months). The methods for measuring the secondary outcomes are described in more detail in the appendix (pp 30–32). Management quality was a prespecified secondary outcome, and will be reported elsewhere.

### Statistical analysis

We planned to recruit 240 health facilities. For compliance with IPC practices, on the basis of estimated compliance of 31% in the control group^8^ and an assumed intracluster correlation coefficient of 0·1, a sample size of 120 facilities in each of the two groups with 115 indications observed in each facility was estimated to provide 80% power to detect an absolute increase of 5·6 percentage points in the intervention group versus the control group at a 5% level of significance. For correct case management, on the basis of an estimate of 52% in the control group and an assumed intracluster correlation coefficient of 0, a sample size of 120 facilities in each of the two groups with four standardised patient visits in each facility was estimated to provide 80% power to detect an absolute increase of 9·0 percentage points in the intervention group versus the control group at a 5% level of significance. These calculations conservatively took no account of the stratified randomisation.

The analysis of the primary outcomes accounted for clustering using a multilevel mixed-effects logistic regression that included facility random effects and stratum fixed effects. A modified intention-to-treat analysis was done for all endpoints, and included all facilities that remained open until the corresponding endline assessment. We report effect sizes as both marginal (absolute) effects and odds ratios. For compliance with IPC practices, analysis was at the level of indication. The primary outcome was a dichotomous outcome that is present if the health worker undertook the correct IPC action corresponding to the indication, as defined in the appendix (pp 29–30). For correct case management, analysis was at the level of standardised patient visit with data pooled across the four cases. The primary outcome was a dichotomous outcome that is present if a standardised patient received correct case management during a visit as defined in the appendix (p 4). For comparison of correct case management by individual case, we used penalised maximum likelihood logistic regression to address the problem of separation within strata.^28^ We also report results from a series of sensitivity checks and prespecified subgroup analyses, including compliance with IPC practices by safety domain (appendix pp 40–49). Although not prespecified, we also examined correct management by standardised patient case. Secondary outcomes measured at the facility level were analysed using ordinary least squares, with the inclusion of stratum fixed effects. The patient experience-of-care score and patient out-of-pocket spending were analysed using a linear mixed-effects model that included facility random effects and stratum fixed effects. We made no adjustment for multiplicity of testing. Missingness was assumed to be at random and missing values were not imputed. Analyses were done with Stata version 14.1. The trial is registered at ISRCTN (ISRCTN93644888).

### Role of the funding source

The funder of the study had no role in study design, data collection, data analysis, data interpretation, or writing of the report.

## Results

**Figure 1:**
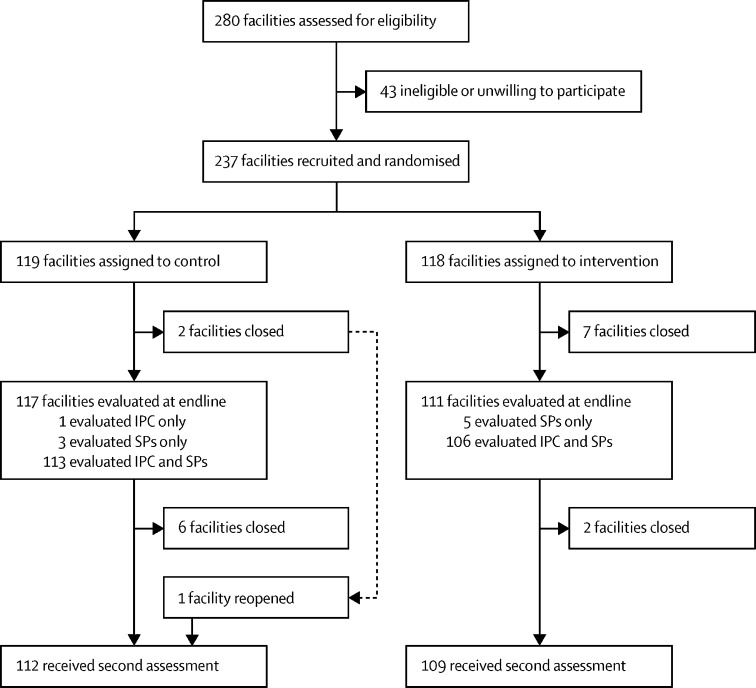
Trial profile At least one provider–patient interaction was observed in 223 facilities (five facilities had no eligible patients) and at least one IPC indication in 220 facilities (three facilities had interactions with no indications). At least one standardised patient case was assessed in 227 facilities. IPC=infection prevention and control practices. SP=standardised patients.

Facilities were recruited from March 7 to Nov 30, 2016. 280 facilities were selected as potentially eligible. After approaching the facilities, 43 (15%) were ineligible or unwilling to participate, leaving a total of 237 facilities participating, of which 118 were randomly assigned to the intervention group and 119 to the control group (figure 1). Compliance with IPC practices was assessed between Feb 7 and April 5, 2018, (mean overall follow-up period of 21·2 months [SD 2·1]; 21·2 months [1·9] in the intervention facilities and 21·2 months [2·2] in the control facilities) in 228 facilities (eight facilities had permanently closed since recruitment and one was closed for renovations). IPC observations in eight (4%) of the 228 facilities resulted in no indications being observed. 29 608 IPC indications were observed (14 366 in the intervention group and 15 242 in the control group) in 5425 provider–patient interactions. Correct management of standardised patients was assessed between May 3 and June 12, 2018 (mean follow-up period of 23·8 months [SD 2·0]; 23·8 months [1·8] in the intervention facilities and 23·8 months [2·2] in the control facilities), in 227 facilities (one facility, assigned to the control group was owned by a private company and served only their employees so standardised patients could not visit undercover). 909 standardised patient visits were done (444 visits in the intervention group and 465 in the control group). The endline SafeCare assessment was done between Oct 12, 2017, and Dec 3, 2018, (mean follow-up period of 23·4 months [SD 1·4]; 23·5 months [1·6] in the intervention facilities and 23·4 months [1·2] in the control facilities) in 221 facilities (a further eight facilities closed down and one facility reopened).

118 (100%) facilities in the intervention group received a quality improvement plan based on a visit from a quality assessor. Fidelity was lower with respect to mentoring visits (mean 3·1 visits [SD 1·5] of five expected) and training sessions (mean 0·6 [0·8] of two expected, although informal training during mentoring visits was generally not recorded). Only two of the 118 intervention facilities received a loan as part of the intervention.

There was no difference in the baseline SafeCare score between the two randomised groups (table 1). The intervention and control facilities were also well balanced with respect to other baseline characteristics. 132 (56%) of the 237 facilities were dispensaries. They were spread across urban, periurban, and rural areas, with the majority (194 [81%] of 237) located outside the commercial capital, Dar es Salaam. Infrastructure and staffing numbers reflected the small or medium size of many facilities. The median number of patient visits per clinician per day was 8·7 (IQR 4·3–16·7) in the intervention group, and 8·2 (3·8–14·2) in the control group. The age and sex of patients observed during IPC observations were similar in the intervention and control groups (appendix p 39). Nine facilities were lost to follow-up (7 in the intervention group and 2 in the control group), although there was no evidence that attrition affected baseline balance between trial groups (appendix p 36). We found no strong evidence of IPC observations being subject to a Hawthorne effect (appendix pp 41–44).

**Table 1:** Baseline characteristics of intervention and control facilities

		**Intervention facilities (n=118)**	**Control facilities (n=119)**
SafeCare assessment score*		41·8% (12·5)	41·7% (12·2)
Partner organisation			
	APHFTA	60 (51%)	59 (50%)
	CSSC	58 (49%)	60 (50%)
Facility level			
	Dispensary	65 (55%)	68 (57%)
	Health centre	35 (30%)	33 (28%)
	Hospital	18 (15%)	18 (15%)
Facility location			
	Inside Dar es Salaam, Tanzania	22 (19%)	21 (18%)
	Outside Dar es Salaam, Tanzania	96 (81%)	98 (82%)
Facility location type			
	Urban	38 (32%)	35 (29%)
	Periurban	32 (27%)	34 (29%)
	Rural	48 (41%)	50 (42%)
Facility opening hours			
	Open 24 h, 7 days a week	72 (61%)	76 (64%)
Staffing and infrastructure			
	Number of medical doctors	0 (1–2)	1 (0–2)
	Number of clinical officers	2 (1–3)	1 (1–2)
	Number of nurses and midwives	2 (1–5)	3 (1–5)
	Number of total staff	14 (9–27)	15 (9–27)
	Number of consulting rooms	2 (1–3)	2 (1–3)
	Number of inpatient admission beds	0 (0–45)	0 (0–30)

Data are mean (SD), n (%), and median (IQR). APHFTA=Association of Private Health Facilities in Tanzania. CSSC=Christian Social Services Commission.

* % of maximum score.

In the intervention group, 8181 (56·9%) of 14 366 indications were met with IPC-practice compliance and in the control group 8336 (54·7%) of 15 242 indications were met with IPC-practice compliance, with an absolute difference of 2·2 percentage points (95% CI −0·2 to 4·7; p=0·071; table 2) and an intracluster correlation coefficient of 0·030. In the adjusted analysis, controlling for age, sex, and indication, IPC-practice compliance was 2·3 percentage points higher (0·3 to 4·4; p=0·028) in the intervention group than in the control group. In the intervention group, 120 (27·0%) of 444 standardised patients received correct case management, compared with 136 (29·2%) of 465 in the control group, with an absolute difference of −2·8 percentage points (95% CI −8·6 to 3·1; p=0·36; table 2) and an intracluster correlation coefficient of less than 0·0001. The results remained similar when we adjusted the analysis for standardised patient fieldworker and case, weighted the data using patient volume, or excluded the 48 (5·2%) of 909 standardised patient visits categorised as detected in the follow-up telephone survey (appendix p 44). Results of the post-hoc subanalyses of IPC-practice compliance by safety domain and correct case management by type of case also showed no significant differences between groups (table 2); prespecified subgroup analyses for the primary endpoints are presented in the appendix (pp 45–46).

**Table 2:** Primary outcomes at endline

	**Intervention facilities**	**Control facilities**	**Absolute percentage point difference (95% CI)**	**OR (95% CI)**	**p value**
IPC compliance*	8181/14 366 (56·9%)	8336/15 242 (54·7%)	2·2% (−0·2 to 4·7)	1·10 (0·99 to 1·21)	0·07
Hand hygiene compliance	361/4201 (8·6%)	295/4454 (6·6%)	1·1% (−0·7 to 2·8)	1·35 (0·84 to 2·17)	0·23
Glove use compliance	2539/3369 (75·4%)	2576/3539 (72·8%)	3·5% (−2·7 to 9·8)	1·22 (0·86 to 1·73)	0·27
Injection and blood draw compliance	4114/4228 (97·3%)	4306/4515 (95·4%)	0·7% (−0·2 to 1·6)	1·61 (0·89 to 2·92)	0·14
Disinfection compliance	16/416 (3·8%)	24/426 (5·6%)	0·1% (−1·6% to 1·7)	1·07 (0·28 to 4·06)	0·93
Waste segregation compliance	1183/2152 (55·0%)	1196/2308 (51·8%)	4·2% (−2·4 to 10·8)	1·19 (0·90 to 1·57)	0·22
Overall correct case management of standardised patients†	120/444 (27·0%)	136/465 (29·2%)	−2·8% (−8·6 to 3·1)	0·87 (0·65 to 1·17)	0·36
Asthma	7/111 (6·3%)	6/116 (5·2%)	1·2% (−5·2 to 7·6)	1·23 (0·42 to 3·55)	0·71
Non-malarial febrile illness	79/111 (71·2%)	85/117 (72·6%)	−1·9% (−13·8 to 10·0)	0·91 (0·50 to 1·64)	0·75
Tuberculosis	23/111 (20·7%)	33/116 (28·4%)	−9·0% (−20·0 to 2·1)	0·60 (0·32 to 1·13)	0·12
Upper respiratory tract infection	11/111 (9·9%)	12/116 (10·3%)	−1·1% (−9·3 to 7·1)	0·89 (0·38 to 2·08)	0·80

Data are n/N (%), unless otherwise specified. For standardised patients, the denominator in the control group varies across cases because one facility received two standardised patients with non-malarial febrile illness. IPC=infection prevention and control practices. OR=odds ratio.

* Assessed in 106 facilities in the intervention group and 114 facilities in the control group.

† Assessed in 111 facilities in the intervention group and 116 facilities in the control group.

**Figure 2:**
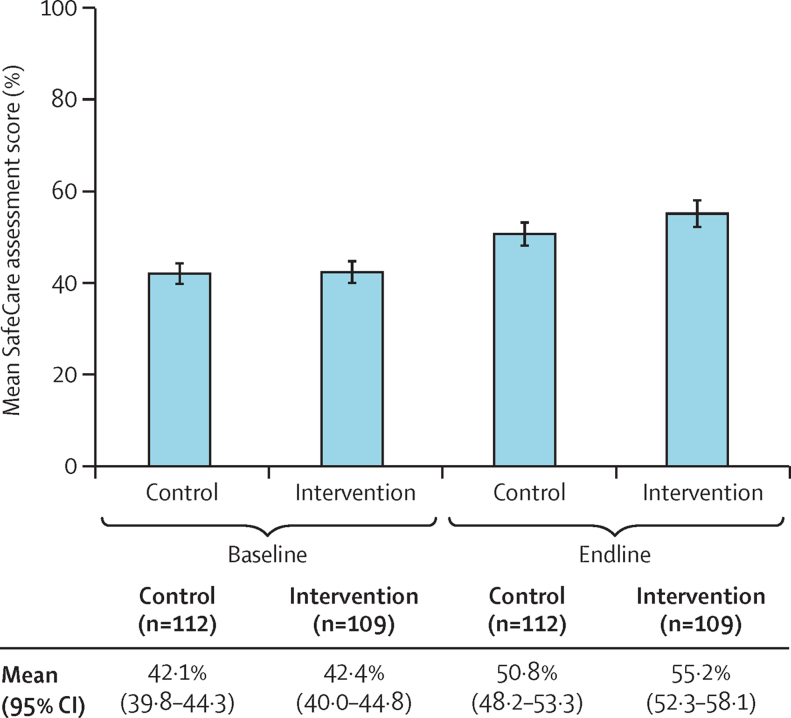
Change in SafeCare assessment score over time in the intervention and control groups Error bars=95% CI.

Facilities in the intervention group had a mean endline SafeCare assessment score of 55·2% (95% CI 52·3–58·1) compared with 50·8% (48·2–53·3) for facilities in the control group (figure 2). The difference was 4·4 percentage points (95% CI 0·9–7·7; p=0·015). The increase in SafeCare score between baseline and endline was 12·8 percentage points (95% CI 10·1–15·4) in the intervention group, compared with 8·7 (6·7–10·7) in the control group, with a between-group difference in score change of 4·0 percentage points (0·8–7·1; p=0·014). The increase in the SafeCare score reflected improvements in a number of service elements: human resource management, patient rights and access to care, risk management, inpatient care, and support services (appendix p 37). At endline, 9 (8%) of 109 intervention facilities and 7 (6%) of 112 control facilities had attained SafeCare level 4, and none in either group had attained level 5.

Table 3 reports the results of the other secondary outcomes. Differences between the trial groups in monthly patient volumes and monthly facility revenue were large and economically meaningful but the point estimates were imprecise, with CIs that included both the possibility of no effect, or a large and important effect. There was no significant difference in patient experience of care and patient out-of-pocket spending.

**Table 3:** Secondary outcomes at endline

	**Intervention facilities**		**Control facilities**		**Difference (95% CI)**	**p value**
	n	Mean (SD)	n	Mean (SD)		
Facility patient visits per month	111	1024 (1447)	117	822 (1050)	145 (−111 to 401)	0·27
Outpatient visits	111	936 (129)	117	735 (903)	145 (−90 to 380)	0·22
Inpatient admissions	111	89 (199)	117	87 (193)	−1 (−34 to 33)	0·97
Facility revenue, US$* per month	105	8833 (18 483)	106	6840 (10 194)	1664 (−2061 to 5389)	0·38
Cash user fee revenue	107	5143 (14 683)	108	3999 (6057)	790 (−2167 to 3748)	0·60
Insurance revenue	107	2850 (6886)	111	2152 (4560)	582 (−771 to 1935)	0·40
Other revenue sources	110	868 (3434)	114	541 (1899)	270 (−387 to 927)	0·42
Patient experience of care†	668	90·8% (8·9)	733	90·7% (8·6)	0·2 (−1·0 to −1·4)	0·72
Patient out-of-pocket spending, US$*	668	5·17 (8·37)	733	4·91 (7·90)	0·13 (−1·41 to 1·68)	0·87

Data are n (number of facilities or number of patients accordingly) or mean (SD), unless specified. For facility-level outcomes, the difference is from an ordinary least squares regression that included stratum fixed effects. The p value is based on robust standard errors. For patient-level outcomes, the difference is from a linear mixed-effects model that included facility random effects and stratum fixed effects.

* Converted from Tanzanian shillings using 2018 World Bank exchange rate, 1 US$=2263·781 TZS.

† % of maximum score.

We carried out an exploratory cross-sectional analysis of our own data to assess associations between SafeCare level and clinical quality. Pooling intervention and control facilities, we found a modest positive correlation between SafeCare level and correct standardised patient management (Spearman's rank correlation coefficient 0·225, p=0·0008), and no association between SafeCare level and IPC-practice compliance (p=0·13).

## Discussion

We did a randomised controlled trial of an intervention package designed to address quality of care in a holistic manner in health facilities in a low-resource setting. The intervention started from a very low base in terms of clinical quality; IPC-practice compliance in the control group at endline was observed in only 55% of indications, and correct management occurred in just 29% of standardised patients. The intervention had a modest positive effect (an increase of 4·4 percentage points) on compliance with SafeCare standards, as measured by the SafeCare assessment score. However, this increase in SafeCare score did not translate into an improvement in clinical quality, with 57% of indications being compliant with IPC practices and only 27% of standardised patients receiving correct case management in the intervention group. The findings are perhaps unsurprising given the small effect of the SafeCare package on SafeCare score results.

Turning to the strengths and limitations of the evaluation, generalisability was enhanced by the study's large scale, wide geographical reach, inclusion of both faith-based and for-profit facilities, and real-world operational conditions. By design, control facilities received a copy of their baseline SafeCare report, but as the report was not accompanied by any explanation or further support, implementers felt it was very unlikely to have led to a substantial quality improvement in most facilities. A key strength of the evaluation was the use of robust measures of clinical quality of care: IPC observation and standardised patients. Process measures such as these directly assess provider behaviour and adherence to established clinical guidelines, and are therefore much more closely related to health outcomes than structural measures. IPC observations were potentially susceptible to the Hawthorne effect,^29^ in which study participants' awareness of being observed causes them to alter their behaviour. However, when we examined the relationship between compliance and order number of patients observed, we found no strong evidence of such an effect (appendix pp 41–43). The use of standardised patients has a particular advantage as a quality measurement tool, allowing comparison across facilities without confounding due to mixing of patients and cases, and allowing facilities to be blinded to measurement and fieldworkers masked to random assignment. A limitation of the study was that the cases presented by the standardised patients needed to have no visible symptoms or require invasive examinations. More generally, in our primary outcomes, it was not possible to capture all types of clinical improvement that might be expected from the intervention (eg, we did not assess inpatient care or emergency referrals). However, the measures were chosen to be core to the intervention scope, and of high priority in public health terms.

We did not do a costing of the intervention, but it is possible to roughly estimate the cost per facility on the basis of the overall SafeCare expenditure for Tanzania, of which the intervention group facilities were part. The total grant was for US$3·9 million for 5 years, and 466 facilities were enrolled. Excluding $158 000 not directly related to the facility-level intervention implies a cost of just over $8000 per facility.

Although no other evaluations of SafeCare-style programmes were identified in private facilities in LMICs, two evaluations of similar accreditation programmes for private hospitals had similar results to the current study. A randomised controlled trial in South Africa^30^ and a non-randomised controlled study in Zambia^31^ both found that the accreditation programme had an effect on accreditation score, but almost no effect on other quality indicators. No other robust studies were identified.^32^ Two randomised controlled trials of social franchising in private facilities in India also found no or weak effect on quality of care.^33^, ^34^ A study of SafeCare in public facilities in Nigeria did not measure clinical quality-of-care outcomes, but found that SafeCare was associated with short-term, but not sustained, increases in some SafeCare standards.^35^

Quality of care was poor in both intervention and control groups. Less than a third of standardised patients in both groups received the correct care for their condition, with particularly low rates for those presenting with asthma and upper respiratory tract infection (table 2). Compliance with IPC practices was particularly low for hand hygiene, disinfection, and waste segregation, although it was high for injection and blood draws (table 2); further results are published elsewhere.^36^ Major deficiencies in quality of care have been reported in similar standardised patient studies in other LMICs,^5^, ^6^, ^37^ and in a similar IPC study from Kenya.^8^ Poor quality of care is unlikely to be explained by facilities being too busy given the estimated median number of patient visits per clinician per day (8·4 per facility).

To explore the reasons for the absence of improvement on clinical quality, we consider the fidelity and quality of implementation, contextual factors, and the functioning of the expected causal mechanisms.^23^ We draw on the process evaluation (to be published separately), which involved a review of administrative data and interviews with facilities, implementing staff, and other stakeholders.

Facility staff were positive about SafeCare. They found the SafeCare standards well conceived and appreciated their holistic nature across the facility, although some smaller facilities perceived the standards as very demanding. The staff praised the clarity of the quality improvement plans, and found implementing staff friendly and helpful, particularly valuing mentorship visits as important for guidance and morale. The main concern of facility staff was the infrequency of mentoring visits, which partly reflected the relatively low intensity of the planned intervention, together with lower than planned implementation: facilities received less than two-thirds of planned mentoring visits and training sessions. Implementation staff explained that a key factor behind lower than expected implementation was delays in receiving their budget from the funder. Implementation staff also highlighted the challenges of reaching some remote facilities, and the reluctance of some facility staff to attend classroom training without compensation. However, this intensity of support is not atypical of SafeCare elsewhere in Africa.

There was particularly low uptake of loans to help facilities pay for any substantial upgrading required, obtained by only two intervention facilities, both for-profit, late in the intervention period. Faith-based facilities generally could not take loans because church property could not be used as collateral. Low uptake in for-profit facilities was said to reflect initial problems faced by PharmAccess in establishing relationships with banks, facilities' poor financial records and credit history, and their concerns about high interest rates, the duration of the application process, and the general economic outlook.

In terms of contextual factors, many facilities felt that the deterioration in Tanzania's economic situation negatively affected facility incomes, which might have discouraged investment, and CSSC facilities reported that government support to faith-based facilities had been reduced. Both intervention and control facilities were exposed to a range of other quality-related activities during the study period. This included regular supervision from district health teams, quality improvement programmes run by other non-governmental organisations often for specific health areas (73% of facilities reported participating in at least one such programme), and the government's star rating assessments. These assessments were based on a similar but more narrow assessment of structural quality than SafeCare, and also involved a quality improvement plan, but no training or mentoring. Nearly all facilities had a star rating assessment during our study period, and facilities were concerned that a poor star rating assessment could risk facility closure. Empanelment of private facilities within Tanzania's social health insurance mechanism, the National Health Insurance Fund, was also increasing over the study period. Although participation in quality-related programmes was balanced between study groups (appendix p 47), it is possible that other support activities dampened the measured effect of SafeCare as both intervention and control facilities responded to other programmes, perhaps partly explaining the improvement of 8·7 percentage points in mean SafeCare assessment score in the control group.

There could also be weaknesses in hypothesised intervention causal mechanisms. The SafeCare approach rests on changes in structural quality (the SafeCare score) leading to changes in care processes. However, evidence has shown that structural quality is often poorly correlated with process quality.^38^ Cross-sectional analysis of our own data provided mixed evidence on this point: we found a modest positive correlation between SafeCare level and correct standardised patient management, and no association between SafeCare level and IPC -practice compliance. At endline, only 16 (15%) of 237 facilities had reached SafeCare level 4 and none had reached level 5; senior SafeCare implementation staff argued that the links between structure and clinical quality are likely to be stronger as facilities progress to these higher levels, when they have the basics in place and focus more on adherence to clinical standards.

The intervention theory also relies on facilities being able to afford to improve standards. Although standards had negligible budget implications, smaller facilities were said to struggle to pay for more qualified staff, or a regular supply of IPC-practice materials. Finally, a key assumption is that facility staff will be motivated to increase compliance with SafeCare standards, either because of intrinsic concern for quality of care, or because they view SafeCare as good business sense. However, the link between SafeCare standards and increased facility income could be tenuous if patients cannot easily perceive quality improvements or if providers do not expect patients to be sensitive to improvements. Stronger external incentives might enhance motivation—eg, if SafeCare scores were directly linked to facility empanelment or reimbursement in social health insurance, or to regulatory penalties.^39^, ^40^ It is important to consider quality improvement as a systems-level issue, requiring not only behaviour change at the facility level, but also better preservice education, coordination across quality improvement mechanisms, and appropriate integration with health financing, procurement, and regulatory systems.

Quality of care was low in both intervention and control facilities, highlighting the importance of developing strategies to improve quality of care at the provider and system levels. However, although the SafeCare intervention led to modest improvements in SafeCare assessment scores, no effect was seen on clinical quality of care. These findings indicate that a higher burden of proof should be placed on policy makers and funders looking to invest in such interventions. Further experimentation and evaluation is clearly needed to identify effective and affordable quality improvement approaches, with possible strategies including increased intervention intensity using digital approaches, more careful selection of facilities for enrolment, creation of stronger financial or regulatory incentives for quality improvement, and increased focus on improving clinical care processes.

## Data sharing

Individual participant data that underlie the results reported in this Article (text, tables, figures, and appendices), after deidentification, along with the study protocols, data collection tools, and analytic code, will be made available immediately following publication without end date, to anyone who wishes to access the data for any purpose. Data will be available at the London School of Hygiene & Tropical Medicine Data Compass site.

## Declaration of interests

This study, through the Health Systems Research Initiative, was partly funded by the UK Government's Department for International Development, which also funded the SafeCare intervention through its Human Development Innovation Fund programme.
