# Supplementary material for: Effect of a multifaceted intervention to improve clinical quality of care through stepwise certification (SafeCare) in health-care facilities in Tanzania: a cluster-randomised controlled trial
Source: Lancet Glob Health. 2021 Aug 4;9(9):e1262–72. doi: 10.1016/S2214-109X(21)00228-X (PMC8370880; doi:10.1016/S2214-109X(21)00228-X)
Supplement: Supplementary appendix [file mmc1.pdf]

THE LANCET
Global Health

Supplementary appendix

This appendix formed part of the original submission and has been peer reviewed. We post it as supplied by the authors.

Supplement to: King JJC, Powell-Jackson T, Makungu C, et al. Effect of a multifaceted intervention to improve clinical quality of care through stepwise certification (SafeCare) in health-care facilities in Tanzania: a cluster-randomised controlled trial. *Lancet Glob Health* 2021; published online Aug 4. http://dx.doi.org/10.1016/S2214-109X(21)00228-X.

Appendix

Contents

Appendix 1

Appendix A: Study sample 2

Selection of study facilities 2

Sample sizes 2

Figure A1: Map of facilities 3

Timeline 3

Appendix B: Data 4

Standardised patients 4

Protocol document for standardised patients 14

IPC observations 26

SafeCare assessment 30

Health facility survey 30

Patient exit interviews 31

Appendix C: SafeCare programme 33

Components 33

Theory of change 35

Appendix D: Additional results 36

Hawthorne effect sensitivity analysis 41

SP detection survey 44

Sub-group analyses 45

Additional quality support 47

Appendix A: Study sample

Selection of study facilities

Eligible facilities were dispensaries and health centres which are members of APHFTA (the Association of Private Health Facilities in Tanzania which represents mainly for-profit facilities), and dispensaries, health centres and hospitals which were members of CSSC (the Christian Social Services Commission which represents most mission facilities). Facilities were ineligible if they refused consent, provided specific services only (e.g. mental health or maternity), or were tertiary hospitals. Facilities were recruited from the Northern, Eastern, Central, Southern and Southern Highlands zones of Tanzania (Lake Zone was excluded because SafeCare had been rolled out there prior to study commencement).

The selection of study facilities was based on an initial long list of 975 private health facilities provided by the implementing partners (462 APHFTA member facilities and 513 CSSC member facilities). We then worked with the implementing partners to select a sampling frame of 280 facilities that potentially met study eligibility criteria. For the CSSC facilities, we selected a random sample of 124 health facilities, stratified by facility type (dispensary, health centre, hospital). For the APHFTA facilities, we were given a list of 156 health facilities that included dispensaries and health centres. Because of the sampling strategy, we do not claim that the study sample is representative of the broader population of health facilities in the study zones.

The partner organisations approached the 280 potentially eligible facilities to confirm eligibility, carry out sensitisation and obtain written informed consent to participate. Of these, 43 declined to participate in the study or were found to be ineligible, such that 237 facilities were recruited at baseline. Facilities were informed at sensitisation that there would be an endline assessment as well as other data collection for both intervention and control arms in order to measure the impact of the SafeCare package.

Sample sizes

Table A1. Sample sizes by survey tool

SURVEY	N	NOTES
Baseline SafeCare assessment	237	All study facilities
Standardised patients	227	9 facilities closed at time of survey visit, consent not sought. 1 facility only serves company employees so was excluded
IPC observations	220	9 facilities closed at time of survey visit, 5 had no eligible patients, 3 had patients but no IPC indications observed
Facility survey	228	9 facilities closed at time of survey visit
Revenue	211	8 additional facilities did not provide data on revenue in interview
Utilisation	228	
Patient exit interviews	224	9 facilities closed at time of survey visit, 4 had no eligible patients
Satisfaction	224	
Expenditure	224	
Endline SafeCare assessment	221	16 facilities shut between baseline and endline SafeCare assessments

Figure A1: Map of facilities

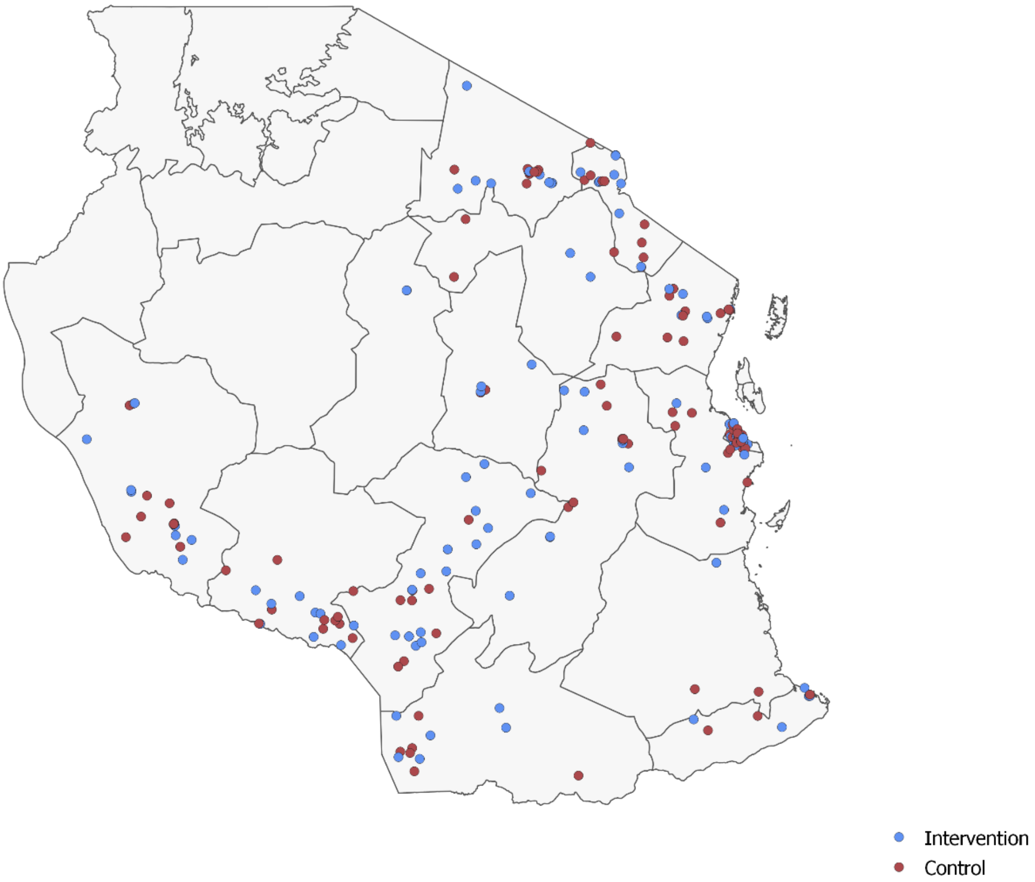

Timeline

		2016				2017				2018			
		Q1	Q2	Q3	Q4	Q1	Q2	Q3	Q4	Q1	Q2	Q3	Q4
Implementation Activities	Baseline SafeCare Assessment												
	QIP delivery												
	Training												
	Monitoring visits												
	Loans disbursed												
	Endline SafeCare Assessment												
Research Activities	Facility survey, IPC observations and patient exit interviews												
	Standardised patients												

Appendix B: Data

Standardised patients

Standardised patient correct management definition

SP case	Initial presentation	Further details given if asked	Correct management
**Asthma**	"I have had a problem with breathing, and last night it became terrible"	Attacks of shortness of breath and wheezing, triggered by exertion, normally at night, lasting 15 minutes to two hours and becoming more frequent over last year	Prescription of inhaled bronchodilator or steroid
**Non-malarial febrile illness**	"I have a fever and I think I have malaria"	Fever and headache lasting three days, joint and muscle pain. Has taken paracetamol for two days.	Malaria test with a negative result, and no antimalarial prescribed or dispensed
**Tuberculosis**	"I have had a cough and it is not getting better"	Three week cough with yellow sputum, no blood, low grade fever, chest pain, night sweats, loss of appetite and weight. Completed seven day course of amoxicillin with no improvement.	Order a sputum test, or refer to a higher level facility
**Upper respiratory tract infection**	"I have a cough and my head and throat hurt"	Symptoms for three days, blocked nose and sneezing, no fever.	No antibiotic prescribed or dispensed

Scripts

Asthma

A STUDY TO EVALUATE THE EFFECTIVENESS OF THE "SAFECARE" MODEL IN TANZANIA

STANDARDISED PATIENT SCRIPT
CASE OF ASTHMA

Opening statement:
Doctor, I have had a problem with breathing, and last night it became terrible.

What difficulties were you having with your breathing?
I was short of breath; I couldn't take a full breath.

What happened last night?
I was at my cousin's place and we were moving around furniture/cleaning. At night I had an attack of breathing problems.

How long was the attack last night?
It was bad for 15 minutes; then I felt a bit better, but didn't feel well for about 2 hours. Even after that I was exhausted.

Were you coughing?
Last night, I was having cough.

How long did you cough for?
Throughout the attack

Did you cough any sputum/mucus?
No.

Were you wheezing/whistling?
Yes, I was last night

Did the attack stop you sleeping?
After the attack I was able to sleep fine

Did you eat anything new last night?

No, I ate rice/ugali/bananas with beans which I often have

Have you had any attacks like this before?

Yes, a number of times, but this is the worst I have had.

Do the attacks wake you up at night?

Yes, sometimes they do

Since when have you had this problem with breathing?

This began one year ago.

Is the shortness of breath constant or does it come and go?

It comes and goes

How often does this happen?

Over the last 3-4 months, it has occurred about once a month. Over the last week this started happening every day.

What brings on the shortness of breath?

It occurs when I am cleaning something, or running a lot or doing any hard work

How long does an attack last?

Earlier it was mild and lasted for only a few minutes. But it has been getting worse over the last 3-4 months, and lasting about an hour.

Is it worse in the morning or evening?

Most of the times I have had attacks it has been evening or night.

Have your lips ever turned blue from struggling to breathe?

No

Have you taken any medication for this problem?

No, never

Is there anything you do to help you cope with an attack?

I get up and walk around

How far can you walk during an attack?

A few metres

Are you breathless even at rest during an attack?

Yes, I still struggle to breathe

Does anyone else in your family have this [breathing] problem?

Yes, my brother also has the same difficulties

Does he take medication for it?

I don't know

Does anyone else in your family have asthma?

I don't know

Does anyone in your family take medication for asthma?

I don't know

Have you ever had a test for asthma?

No

Did you have this breathing problem as a child?

I don't remember, but my mother says I used to cough a lot.

Do you have fever?

No.

Do you have chest pain?

No.

Are you losing weight?

No.

Have you lost your appetite?

No.

Are you having night sweats?

No.

Have you had any sore throat, cold, sneezing or stuffiness?

No.

Do you smoke?

No.

Do you drink?

No

Are you allergic to any medicines?

No

Do you have any other problems?

No

When was your last period?

About two weeks ago

Are you/could you be pregnant?

No

A STUDY TO EVALUATE THE EFFECTIVENESS OF THE "SAFECARE" MODEL IN TANZANIA

STANDARDISED PATIENT SCRIPT
CASE OF NON-MALARIAL FEBRILE ILLNESS

Opening statement:
Doctor, I have a fever and I think I have malaria

Why do you think it's malaria?
Because I have a fever and a headache

What are your symptoms?
I have a fever and headache.

Which symptom started first?
They started at the same time

How long have you had these symptoms for?
For three days

Is the fever constant or does it come and go?
It comes and goes

Does the fever go up and down?
Yes

When you have a fever is it very high?
Sometimes high, sometimes low

Have you been able to eat and drink?
Yes, I ate a small breakfast and drank some water

Have you had any vomiting or diarrhoea?
No.

Have you taken any medicines?
Just panadol

For how long?
Two days

Have you taken a malaria test?
No

When was the last time you had malaria?
About one year ago

Have you travelled recently?
Yes, I've been to Tanga/Morogoro/Mtwara/Mwanza

Have you had difficulty breathing?
No

Have you had any wheezing?

No.

Have you had any muscle or joint pain?

Yes, my muscles and joints ache

Do you have chest pain?

No.

Do you have a cough?

Yes, a little coughing

Have you had a cold, sneezing, sore throat or stuffiness?

No

Does the cough produce sputum/mucus?

No

Do you have any pain on coughing?

No

Have you had any fainting or convulsions?

No.

Do you feel dizzy?

No

Do you smoke?

No.

Do you drink?

No.

Are you allergic to any medicines?

No

Do you have any other problems?

No

When was your last period?

About two weeks ago

Are you/could you be pregnant?

No

A STUDY TO EVALUATE THE EFFECTIVENESS OF THE "SAFECARE" MODEL IN TANZANIA

STANDARDISED PATIENT SCRIPT
CASE OF TB

Opening statement:
Doctor, I have had a cough that is not getting better.

How long have you had a cough for?
About 3 weeks

Do you cough up mucus/sputum?
Yes, some yellow mucus

Is there blood in the sputum?
No

Have you seen a doctor already?
Yes, and he gave me some medicines

Which health facility?
[Name facility in another town]

How long did you have your cough for when you saw the doctor?
One week

Did the doctor do any tests?
Yes, he did a malaria test but it was negative

What medicine did you take?
Amoxicillin

How long have you been taking the medicine for?
One week

Did you finish all the medicine?
Yes

Have your symptoms improved?
No, they haven't gone away at all.

Have you ever been tested for TB?
No

Have you ever been diagnosed with TB?
No

Has anyone in your family had TB?
No

Has anyone in your family had a cough like this?
No

Have you had any contact with any TB patients?
No

Do you have the cough throughout the day?

Yes, all day, but it comes and goes

Have you had a fever?

Yes, some fever

Was your fever very high?

Not especially

Do you have chest pain?

Yes.

Whereabouts in your chest is the pain?

All over

Have you lost your appetite?

Yes.

Have you had difficulty breathing?

No.

Have you had any wheezing?

No.

Are you losing weight?

Yes.

How much?

I don't know, just a little. My clothes feel a little looser.

How much did you weigh the last time that you weighed yourself?

I can't remember

Are you having night sweats?

Yes.

Have you had any throat pain or upper respiratory symptoms (cold, sneezing, stuffiness)?

No.

Do you smoke?

No

Do you drink?

No

Do you have diabetes?

No

Have you had diabetes in the past?

No

Have you been tested for diabetes?

No

Have you ever taken an HIV test/do you know your HIV status?

No

Are you allergic to any medicines?

No

Do you have any other problems?

No

When was your last period?

About two weeks ago

Are you/could you be pregnant?

No

A STUDY TO EVALUATE THE EFFECTIVENESS OF THE "SAFECARE" MODEL IN TANZANIA

STANDARDISED PATIENT SCRIPT

CASE OF URTI

Opening statement:
I have a cough and my head and throat hurt

How long have you had these symptoms for?
3 days

Which symptom started first?
They started at the same time

Do you have a fever?
No

Have you taken any medications?
No

Do your symptoms get worse at night/change through the day?
No, they are the same at day and night

Did you cough any sputum?
Yes, a little

Is there blood in the sputum?
No

Do you have a running nose?
A little bit

Do you have any sneezing?
Yes

Do you have a blocked nose?
Yes, I feel a bit stuffy

Do you have any allergies?
No

Do you have chest pain?
No.

Have you lost your appetite?
No.

Do you have pain on swallowing?
Yes

Have you had difficulty breathing?
No.

Have you had any wheezing?

No.

Are you losing weight?

No.

Are you having night sweats?

No.

Do you smoke?

No.

Do you drink?

No

Are you allergic to any medicines?

No

Do you have any other problems?

No

When was your last period?

About two weeks ago

Are you/could you be pregnant?

No

Protocol document for standardised patients

Standardised patients in the evaluation of SafeCare in Tanzania

Standardised patients (SPs) were used to measure process quality of care, the primary outcome in the evaluation of the 'SafeCare' model of quality improvement in Tanzania. The study is an RCT design, with 237 private health facilities enrolled at baseline (118 intervention, 119 control). SPs were used at endline only. We asked consent for SPs during an earlier visit to the facilities to conduct survey / observation work.

Development process

The tools and protocols in this document were developed through the following steps:

1) A systematic literature review was carried out in November-December 2016. The review identified published examples of the use of covert standardized patients to evaluate all aspects of clinical care. These were drawn upon as guidance for what was feasible and ethical in SP studies.
2) Workshop in Dar es Salaam in January 2017. The workshop was attended by the study team, representatives of PharmAccess International and Tanzania, implementing partners for the SafeCare intervention (APHFTA, CSSC and MCF), and clinical specialists from the Ifakara Health Institute (IHI). The workshop identified the aspects of process quality of care which were most likely to be affected by SafeCare, and the best ways of measuring these.
3) Review of protocols. The study team reviewed the tools and protocols available for two SP studies carried out in India, one examining TB care[1], the other asthma, angina and childhood diarrhoea[2].

SP case choice rationale

A shortlist of conditions was drawn up based on (i) a literature review of use of standardized patients in LMICs, and (ii) conditions reported to be frequently treated in SafeCare facilities in Tanzania. Each condition on the shortlist was then assessed for inclusion on the basis of five criteria:

1. Evidence for treatment: is there clinical evidence (preferably national standard treatment guidelines) by which to define correct treatment or management? This was a prerequisite for consideration.
2. Clinical and public health significance: does recognition and correct treatment of the condition have an important public health role, or is it a serious clinical emergency?
3. Frequency in study facilities: is the condition commonly enough seen in study facilities that correct recognition and treatment is feasible, and it will not arouse suspicion?
4. Risk to fieldworkers and ethical considerations: will the case necessitate practices which expose the fieldworker to hospital-acquired infection, invasive examinations or a life-changing diagnosis?
5. Falsifiability of symptom and ease of diagnoses: can the symptoms be easily falsified by fieldworkers and will the provider be able to make a diagnosis on the basis of those symptoms during a single consultation with limited laboratory testing?
6. Universal applicability: can the condition be diagnosed or treated, or an appropriate referral made, at all facilities in the SafeCare study?

The assessment by the study team is summarised in Table B1 below. As a result, four conditions were selected as most appropriate for SPs in our study: upper respiratory tract infection (URTI), suspected malaria/non-malarial febrile illness, TB and asthma.

Table B1: Standardised patient cases considered for SafeCare evaluation

Case/condition	Clinical and public health significance	Frequency in study facilities[1]	Risk to fieldworker and ethical considerations	Falsifiability of symptoms and ease of diagnosis	Universal applicability
**Included:**					
**Asthma**	Some (not infectious, can be life-threatening)	Low (40/234)	Low- blood tests only to exclude other conditions	Good- can report distinctive breathing difficulties	Yes
**Suspected malaria**	High (life-threatening, infectious, resistance)	High (221/234)	Some- reduced risk with fingerprick testing with single-use lancets	Good- cyclic pattern of fever means no fever required at consultation	Yes
**TB**	High (underdiagnosed, infectious)	Low (assumed)	Low- X-ray required but not in facility	Good- history of cough and weight-loss, cough need not produce blood	Yes
**Upper respiratory tract infection**	High (antimicrobial stewardship)	High (178/234)	Low- blood tests only to exclude other conditions	Good- generic symptoms of headache, coughing and running nose	Yes
**Excluded:**					
**Angina**	Limited (life-threatening, not infectious)	Low (assumed)	Low- blood tests only to exclude other conditions	Limited- angina patients typically appear seriously unwell	Yes
**Child (any condition, absent)**	High (often infectious, significant morbidity)	High (assumed)	Low- child is absent	Poor- attending health facility without child not a cultural norm in Tanzania	Yes
**Child (any condition, present)**			High- child SPs cannot give consent to study participation	Limited- would need to train children	Yes
**Depression**	High (significant morbidity, underdiagnosed)	Low (assumed)	Low- blood tests unlikely	Limited- unlikely to be recognised non-specialist facilities	Yes
**Diabetes**	High (significant morbidity, underdiagnosed)	Low (65/234)	Some- blood glucose test requires fingerprick	Limited – symptoms can be falsified but not blood glucose levels	Yes
**Diarrhoea**	High (significant morbidity, infectious)	High (188/234)	Low- blood tests only to exclude other conditions	Poor- can't provide stool sample	Yes
**Family planning client**	High	Variable (up to 480 visits per month)[2]	Some- pelvic exam can be refused	Good- no symptoms needed	No[2]
**HIV testing**	High (infectious)	Medium (85/234)	High -could be mitigated by testing fieldworkers before study	Good- no symptoms needed	Yes
**Hypertension**	High	Medium (100/234)	Low-blood tests only to exclude other conditions	Poor- will not be hypertensive	Yes
**Injuries and accidents**	High	Medium (81/234)	Low- blood tests unlikely	Poor- difficult to falsify injuries	Yes

**Pregnancy testing**	Limited (interest in antenatal care, not pregnancy testing)	High (191/234)	Low- blood tests unlikely	Limited- symptoms easily falsifiable but urinalysis will be negative	Yes
**Skin diseases**	Limited	High (123/234)	Low- blood tests unlikely	Poor- difficult to falsify skin complaints	Yes
**STI**	High (significant burden, infectious)	High (147/234)	Some- pelvic/genital exam, difficult to refuse	Limited- can report pain and discharge but can't falsify visible symptoms	Yes
**UTI**	Limited	High (227/234)	Low- blood tests unlikely	Good- painful and frequent urination	Yes
**Worms**	High (significant burden)	High (147/234)	Low- blood tests unlikely	Poor- can't provide stool sample	Yes

[1]Study facilities complete a situational analysis (SA+) form when joining the study. Facilities can choose up to ten conditions from a predefined list as the ones most commonly diagnosed or treated. Frequencies listed are the number of facilities which list a given condition as one of their 'top ten'. Data are available for 234 of 237 study facilities.
[2]92 facilities reported a non-zero number family planning clients per month (averaged over the last six months) on the SA+. 60 reported zero clients, and 83 that the question was not applicable. Data are available for 235 of 237 study facilities.

Blinding and consent

Facilities were asked to give consent to covert SP visits during the first (overt) phase of fieldwork, but not be given any further details. In order to minimise the risk of SP detection, details of SP cases and their correct management were not be shared with the PharmAccess Tanzania office, or implementing partners APHFTA and CSSC.

Expected management

Expected management covers the taking of medical history, giving correct treatment or referral, and giving appropriate advice. Expected management is based on Tanzanian standard treatment guidelines[3], MAQARI SP manual[2] and Heymann (2015)[4]. The expected management for each case is given in Table B2 below.

Table B2: Expected management of SP cases

Case	History	Treatment/referral	Advice
**Asthma**	• Duration and frequency of breathing difficulties?<br>• Chest pain?<br>• Family history?<br>• Childhood history?<br>• Medications tried?<br>• Allergic triggers?<br>• Presence of fever?<br>• Time of day of attacks?<br>• Smoking?<br>• Weight loss?	• Salbutamol or beclamethasone inhaler	• education on asthma diagnosis<br>• education on allergic triggers<br>• explain use of inhaler
**Non-malarial febrile illness**	• Duration of fever?<br>• Grade of fever?<br>• symptoms of severe malaria?<br>• Coughing?<br>• Wheezing?<br>• weight loss?<br>• muscle pain?	• Blood test for malaria (blood slide or RDT)<br>• Negative result<br>• No prescription of antimalarial	• Advise return in case of severe malaria symptoms
**TB**	• Duration of cough?<br>• Productive cough?<br>• Wheezing?<br>• Blood in sputum?<br>• Doctor consulted and medications tried?<br>• Weight loss?<br>• Loss of appetite?<br>• Presence of fever?<br>• Night sweats?<br>• Chest pain?<br>• HIV status?<br>• Diabetes?<br>• Personal and family history of TB?<br>• Smoking?<br>• Alcohol?	• Referral for x-ray or sputum microscopy	• Explain treatment is free in public facilities
**URTI**	• Duration of symptoms?<br>• Presence of fever?<br>• Breathing difficulties?<br>• Wheezing?<br>• Night sweats?<br>• Chest pain?<br>• Weight loss?	• No antibiotics	• Explain viral infection will cure without treatment

Correct management indicator

As a primary outcome of quality of care, we define a binary indicator of 'correct management' for each SP case. To fulfil correct management, the provider must carry out all 'actions required' and no 'actions precluding' as set out in Table B3.

Table B3: Correct management indicator

Case	Actions required for correct management	Actions precluding correct management
**Asthma**	• Prescription of salbutamol inhaler	
**Non-malarial febrile illness**	• Blood test for malaria (blood slide or RDT)<br>• Negative result	• Prescription of antimalarial
**TB**	• Referral for x-ray or sputum microscopy, or to a facility which can diagnose TB	
**URTI**		• Prescription of antibiotics

Harm minimisation

All fieldwork contains inherent risks, but SP studies expose fieldworkers to additional risks by asking them to pose as real patients. SP cases and training must be designed to minimise these risks. The major risks identified by the study team and MAQARI SP manual[2], and steps taken to reduce them, are detailed below.

1. **Exposure to airborne pathogens in facility**. There is little that can be done to reduce exposure of SPs to respiratory pathogens when waiting inside a facility. More serious respiratory infections, such as TB, are not treated in small clinics, and are treated in separate outdoor clinics in larger facilities. It is therefore thought that the risk of an SP contracting a serious respiratory infection from this work is minimal. This study was conducted in 2018 before the emergence of COVID-19.

2. **Exposure to surface pathogens in facility**. During training, SPs were educated about the pathogens that remain on surfaces inside facilities. They were informed of the importance of hand hygiene after the end of the facility visit, and supplied with alcohol hand gel.

3. **Exposure to pathogens on thermometers**. SPs were trained to avoid having temperature taken with an unsterilized oral thermometer. Training included:
    a. Recognition of when thermometer may not have been sterilized
    b. Asking provider whether thermometer has been sterilized
    c. Asking provider to use sterilized thermometer/sterilize thermometer before use
    d. Refusing to have temperature taken orally if thermometer not sterile
    e. Revealing identity as SP if refusal not accepted

4. **Exposure to pathogens through injections.** SPs were trained to avoid all injections, IV fluids and other parenteral administration of medications. Training included:
    a. Recognition of terms provider may use to indicate they plan to give SP injection
    b. Recognition of provider actions which indicate imminent injection (e.g. preparing needle)
    c. Refusal of injections on grounds of extreme needle phobia "I cannot have injections, the last time I received an injection I lost consciousness"
    d. Refusal of injections on grounds of cost "I do not have the money to pay for an injection with me today, I don't want it"
    e. Refusal of injections on grounds of fasting "I am fasting so I may not have an injection"
    f. Revealing identity as SP if refusal not accepted

5. **Exposure to pathogens through blood draws**. SP cases have been chosen to minimise likelihood of blood tests, with the exception of the malaria case, which requires a finger-prick blood sample. SPs were trained to avoid having blood drawn except from the fingertip with a single-use, sterile lancet for a malaria test or blood glucose test. Training included:

a. Asking provider whether a diagnostic test requires blood to be taken
b. Asking provider where blood will be taken from
c. Recognising a single-use sterile lancet
d. Refusal of blood draw on grounds of extreme needle phobia "I cannot have injections, the last time I received an injection I lost consciousness"
e. Refusal of diagnostic tests on grounds of cost "I cannot do these tests; I can't pay for them"
f. Refusal of diagnostic tests on grounds of inconvenience "I cannot stay and have these tests, I have to meet with someone soon"
g. Leaving the facility and paying fees without having diagnostic tests if refusal not accepted
h. Revealing identity as SP if refusal not accepted

6. **Unnecessary exposure to ionising radiation.** SPs were trained to avoid all X-rays. X-rays are only likely to be offered to TB SPs, and only 37 of 237 study facilities can offer X-ray imaging, so this risk existed in a small number of cases. If offered, SPs refused on grounds of cost.

7. **Administration of unnecessary/harmful medications**. SPs were trained to avoid all medications:
    a. Parenteral administration was avoided as outlined above (4)
    b. SPs were trained to give all dispensed medications to supervisor as soon as reasonably possible, and that they must not under any circumstances take medications. The medications were stored and returned to study team for quality control testing
    c. If provider offers oral medication in facility, SP was trained to refuse on grounds of fasting, and say that they can take it in the evening.
    d. If fasting will not be believed, refused on grounds of cost
    e. SPs must reveal identity rather than take any medication

8. **Invasive physical examinations.** SP cases have been chosen to minimise the likelihood of invasive physical exams. SPs were trained to refuse pelvic/genital exams and any other examination or procedure they do not feel comfortable with, and to reveal their identity as an SP as a last resort if necessary.

9. **Admission to facility.** SPs were trained to avoid being admitted to the facility as an inpatient. They refused to be admitted on grounds of inconvenience, saying they need to return to where they live and will seek medical attention there. If this explanation was not accepted, they were to reveal their identity as an SP.

10. **Diagnosis of previously undetected condition.** There is a risk that SPs may be diagnosed with a genuine medical condition during the course of their work, as a direct result of investigations carried out in study facilities. SP cases have been designed to minimise this risk. SPs were only be recruited if they self-report good health and did not report any underlying conditions. Specific scenarios are outlined below:

    a. HIV: No SP will be diagnosed as blood cannot be taken except for finger-prick samples for malaria or glucose.
    b. Malaria: If SP tests positive for malaria, they must report this to the supervisor and take a second RDT. If this is also positive the SP/supervisor should purchase AL for treatment.
    c. Hypertension: SPs are likely to have blood pressure measured frequently during the study period and may be informed they are hypertensive.

11. **Abuse/harassment at facility due to detection of SP**. During the first (overt) round of field work, facilities were informed of the use of SPs in the study and asked to give their consent. SPs were only be sent to facilities where consent has been given for the use of SPs. The facility was asked to ensure that the manager, duty manager or supervisor has been informed of SPs. A letter was be given to facilities explaining the use of SPs, to be put on file in the facility along with a copy of the ethical approval. If the identity of an SP is revealed, or the SP needs to reveal their identity to avoid harm they should:

a. Explain that they are a fieldworker from the SafeCare/LSHTM/IHI study
b. Thank the provider for their time, pay any outstanding fees and leave the facility
c. If challenged, show copies of letter and ethical approval, and tell provider the facility should also have them on file
d. Provide the contact details of a member of the study team if the provider has further queries

Dispensed medications

SPs must give all medications which are dispensed to them to their supervisor after they have completed details on exit questionnaire. The supervisor checked medication against completed questionnaire to ensure that the correct number of items has been returned. All medications from that visit were sealed in a plastic bag and labelled with the date, facility name and code, fieldworker name and SP case. The supervisor was responsible for the safe-keeping of medications until the team returned to IHI, or a researcher visited the field and collects the medication.

Petty cash and reimbursements

Supervisor was in charge of petty cash book. At the beginning of each working day/week, the supervisor made sure each SP has enough cash to pay for all planned facility visits (TSH 25,000 for APHFTA facilities and TSH 15,000 for CSSC facilities), and recorded any petty cash that needed to be dispensed in advance. Total fees paid were recorded in petty cash book as per SP questionnaire for each facility visit. SP was reimbursed for fees paid, minus advance.

SP detection follow-up

All facilities were followed up to assess whether an SP visit was detected or suspected. Follow-up was conducted by telephone.

Process for obtaining telephone number of owner/facility:

- Record number(s) provided in SA+ and print on facility consent form.
- Ask owner/provider to confirm that this is the correct number when consenting for fieldwork, or to provide an alternative number (or email address if this is not possible).
- If facility/owner is not reachable through number provided in SA+ or at facility, ask APHFTA/CSSC to provide details or make contact on our behalf.

Timing:

- After all four SP visits have been confirmed as completed by the supervisor, wait at least one week until making follow-up call.
- The maximum time between completion of SP visits and follow-up call should be four weeks.

Call script:

"Hello, my name is [name] and I'm calling from Ifakara Health Institute. Am I speaking to/please may I speak to [name of provider/owner/manager]?"

- If not available, ask for a suitable time to call back and leave contact details, or ask for alternative number if cannot be reached at that number.

Once confirmed speaking to provider/owner/manager:

"I am calling about the research we have been doing in your facility. Do you have time to answer a few questions? You may recall that we planned for standardised patients to visit your facility and pose as real patients. I am calling to confirm that those visits have taken place in the past few weeks. Did you or your staff have any suspicion that any patients you saw in the last four weeks were not genuine patients? Please can you give me some details?"

**Suspected SP**	
Date of visit	
Time (can be approximate e.g. morning, evening)	
Patient age (approximate if necessary)	
Patient gender (1= male, 2= female)	
Complaint of patient (any details remembered)	
At what stage did you/your staff become suspicious? (1= immediately at start of consultation, 2= during consultation, 3=after end of consultation)	
Did you confront the patient? (1=yes, 2=no)	
Did you treat the patient differently due to suspicions? (1=yes, 2=no)	
If yes, in what way? [probe- e.g. sent away, didn't prescribe medications]	

[Researcher only] Confirmed SP detection? (1= yes, 2=no)	
[Researcher only- if SP correctly detected] SP case type (1= Asthma, 2=NMFI, 3=TB, 4= URTI)	

- We should not confirm with providers whether their suspicions are correct, or give details of SP case types. SP fieldwork will be ongoing for three months and we need to avoid the risk of information being passed to other facilities.

Recruitment

Numbers: We planned to employ 16 SPs organised into four teams of four, and one supervisor for each team. We aimed to recruit and train 30 fieldworkers to allow for attrition and selection.

Person characteristics and appearance: All scenarios can be acted by men or women in their twenties or thirties.

Health: We aimed to recruit SPs who are in good health to avoid other complaints misleading providers. SPs were be asked if they have been diagnosed with asthma, TB or any serious chronic condition in the past, and were excluded if it was decided that the condition will put them at risk or mislead providers during the SP scenario.

Background: SPs should not have a clinical background.

SP training

SP case development

The main purpose of the SP training was to teach SPs about the case they are meant to portray, and how they should go about doing this. SPs were told about the conditions they are acting, and the symptoms the patient would and would not have. SPs were trained to only give information that is asked for by a care provider, and not to deliberately give more information to 'help' the provider along the way to a diagnosis. SPs practised coming up with answers for unexpected questions, so that they are prepared to give an answer if a provider asks about a symptom or lifestyle factor that the study team did not anticipate when producing scripts.

Background story

Time during training was dedicated to developing background stories for the SP characters. SPs were be trained to portray people from a lower middle class demographic group, to match the type of clients expected at small private providers, and were told what job and education level their character has. SPs were trained to dress according to this character, and to adapt their dress to different areas of the country where necessary.

SPs were given examples of how to explain their attendance at a facility where they are not recognised, and trained to respond to questions about where they are staying and where they are from. The basic backstory, which can be adapted according to SP and setting, is that the SP is visiting to buy cash crops or sell second hand goods. This allows SPs to explain why they are in the area without requiring them to have local knowledge. SPs worked in groups to develop the 'personality' of their SP, working out how their character would respond to different behaviours from providers. This reduced heterogeneity in the portrayal of SP cases across different fieldworkers.

Role play

SPs practised acting their cases in pairs, taking turns to play provider and SP. SPs were given a number of scenarios to allow them to practise their response in different circumstances, for example, refusing an injection, or when asked about a symptom that isn't mentioned in the SP script. Members of the study team also acted as 'provider' to check the progress and portrayal of SPs.

Exit questionnaire

SPs practised completing the exit questionnaire when role-playing SP scenarios. Members of the study team validated exit questionnaires during roleplay for each SP, observing the interaction and also completing the questionnaire, then feedbacking to the SP on any discrepancies.

Risk mitigation strategies

SPs were taught about sources of infection in health facilities and other risks to their safety, and trained to recognise and anticipate when they might be at risk (see Harm minimisation section). Emphasis was placed on refusing care assertively, and revealing status as an SP rather than accepting any potentially harmful practice.

Dress rehearsal

In the final stages of training, SPs had a 'dress rehearsal' at a local facility. The study team approached local facilities that were not part of the study and ask for consent to send SPs to them for training. SPs carried out visits to these facilities in pairs, with a second fieldworker posing as a partner or relative to give feedback, following procedures exactly as they should for fieldwork, including completing the exit questionnaire. A second dress rehearsal was arranged if necessary.

Timetable

Week		Monday	Tuesday	Wednesday	Thursday	Friday	Saturday
1	Morning	Introduction to the study and icebreakers	Role-play practising	Test: SP case knowledge	Background character development	Safety and risk minimisation	Introduction to questionnaire
	Afternoon	Introduction to the SP cases	Role-play practising	Role-play practising	Background character development	Test: safety	Practising with questionnaire
2	Morning	Test: SP case knowledge and safety	Study team validations	Study team validations	Dress rehearsals and videoing	Dress rehearsals and videoing	Dress rehearsals and videoing
	Afternoon	Practising with questionnaire	Logistics: transport and accommodation	Logistics: money and medications	Dress rehearsals and videoing	Dress rehearsals and videoing	Dress rehearsals and videoing

Questionnaire completion

SP data collection was a self-administered smartphone questionnaire immediately after the end of the interaction, verified by the supervisor at the end of the day. SPs were trained to find a suitable location to complete the questionnaire after leaving the facility.

Facility approach

Facilities in town: SPs should walk to the facility. If the facility is a long distance from accommodation, public transportation can be used, or SP can be dropped by an IHI vehicle at least 500m away and approach on foot. Use of IHI vehicles should be limited in small communities to avoid drawing attention to the presence of fieldworkers.

Facilities outside of town: If facility can be reached by bus from the nearest town, SP should travel this way. If not feasible, SP should take taxi (if available at reasonable cost) or be transported in IHI car. When IHI cars are used for transportation to facility, SPs should be dropped at least 500m from facility and approach on foot. A pick-up point should be agreed at this time.

SP teams

SPs were in four teams of four, each with one supervisor, giving a total team size of eight. Teams visited different regions of the country. Each team had one male and one female SPs trained to portray two of the four scenarios, who were be randomly assigned to facilities. Assignment to facilities was stratified as per recruitment.

Timing of facility visit

Visits were made 10am-4pm, to avoid the busiest outpatient clinic times (typically early morning and evening), and night hours where a reduced service operates and some facilities are closed. Visits were planned for Saturday and Sunday only if a full service runs on those days. The visiting time is left flexible between these hours to allow multiple visits per day in towns and cities, and to allow for travel time to remote facilities.

1. Satyanarayana S, Kwan A, Daniels B, Subbaraman R, McDowell A, Bergkvist S, et al. Use of standardised patients to assess antibiotic dispensing for tuberculosis by pharmacies in urban India: a cross-sectional study. The Lancet Infectious Diseases. 2016;16(11):1261-8
2. Mohanan M, Das V, Tabak D, Chan B, Holla A, Das J. Standardized patients and the measurement of rural healthcare quality: Field guide, manual and sample instruments: MAQARI; 2012.
3. United Republic of Tanzania: Standard treament guidelines and essential medicines list. 4th ed: Ministry of Health and Social Welfare; 2013.
4. Heymann DL. Control of communicable diseases manual: American Public Health Association; 2015.

IFAKARA HEALTH INSTITUTE research | training | services

PharmAccess FOUNDATION

LONDON SCHOOL of HYGIENE &TROPICAL MEDICINE

A STUDY TO EVALUATE THE EFFECTIVENESS OF THE “SAFECARE” APPROACH IN TANZANIA

Observation Tool

A: GENERAL DETAILS (ALL SITES)

1. Field Interviewer ID: ____ ____

2. Facility ID: ____ ____ ____

3. Date (DD/MM/YY): ____ ____/____ ____/____ ____

4. Site: □ Consultation room □ Injection room □ Dressing room □ Laboratory

5. HCW ID: ____ ____

6. Patient consent: □ Yes □ No

7. Red bin in room: □ Yes □ No

8. Red bag in red bin: □ Yes □ No

9. Yellow bin in room: □ Yes □ No

10. Yellow bag in yellow bin: □ Yes □ No

11. Start time: :

12. End time :

13. Patient ID: ____ ____ ____ ____

14. Patient gender: □ Male □ Female

15. Patient age (approximate): □ <5 □ 5-17 □ 18-29 □ 30-49 □ 50+

16. Observation result: □ Completed □ Partially completed

17. If partially completed, give reason: □ Ended by provider □ Ended by patient □ Ended by interviewer □ Other (specify): ____________________

B: HAND HYGIENE (CONSULTATION OR DRESSING)

18. Are gloves used?
Yes □
No □

If yes:
19. Are gloves…. □ New □ Reused □ Cannot say
20. Hand hygiene directly before gloves: □ HR □ HW w/soap □ HW no soap □ None □ Cannot say
21. Hand hygiene directly after gloves: □ HR □ HW w/soap □ HW no soap □ None □ Cannot say

22. Patient contact or exam (tick all that apply):
□ Touching skin
□ Cleaning body fluids
□ Mouth or throat exam
□ Nose exam
□ Ear exam
□ Eye exam
□ Wound cleaning or dressing
□ Wound exam
□ Stiches
□ Preparation of medicine
□ Inserting a suppository
□ Vaginal, male genital or rectal exam
□ Listening to chest
□ Handling container of body fluid
□ Taking temperature

□ Other (specify): ____________________

□ No patient contact and no exam (skip to 31)

23. Hand hygiene before contact or exam:
□ HR
□ HW w/soap
□ HW no soap
□ None
□ Cannot say

If yes:
24. Hand hygiene took _______ seconds
25. Hand hygiene with gloves on: □ Yes □ No
26. Drying method: □ Clean disposable towel □ Nothing □ Reused towel or clothes □ Cannot say

27. Hand hygiene after contact or exam:
□ HR
□ HW w/soap
□ HW no soap
□ None
□ Cannot say

If yes:
28. Hand hygiene took _______ seconds
29. Hand hygiene with gloves on: □ Yes □ No
30. Drying method: □ Clean disposable towel □ Nothing □ Reused towel or clothes □ Cannot say

C: WASTE SEGREGATION AND DISINFECTION (CONSULTATION OR DRESSING)

31: Thermometer used: □ Yes □ No

If yes:
32. Type □ Standard □ Infra-red
33. Disinfection (before or after use):
□ Disinfected with rubbing alcohol/bleach
□ Not disinfected, but cleaned
□ Not disinfected, not cleaned
□ Left in disinfectant
□ Cannot say

34. Stethoscope used: □ Yes □ No

If yes:
35. Disinfection (before or after use):
□ Disinfected with rubbing alcohol/bleach
□ Not disinfected, but cleaned
□ Not disinfected, not cleaned
□ Cannot say

36. Tongue depressor used: □ Yes □ No

If yes:
37. Type: □ Plastic □ Wooden □ Metallic
38. Segregated….
□ Black or blue bin □ Yellow bin
□ Red bin □ Other bin
□ Disinfected with rubbing alcohol/bleach
□ Not disinfected, but cleaned
□ Not disinfected, not cleaned
□ Cannot say

39. Otoscope used: □ Yes □ No

If yes:
40. Disinfection (before or after use):
□ Disinfected with rubbing alcohol/bleach
□ Not disinfected, but cleaned
□ Not disinfected, not cleaned
□ Cannot say

41. Gloves used: □ Yes □ No

42. If yes:
Segregated….
□ Black or blue bin □ Yellow bin
□ Red bin □ Other bin
□ Left on hands
□ Left outside
□ Cannot say

43. Swabs/gauze/bandages used on patient: □ Yes □ No

44. If yes:
Segregated….
□ Black or blue bin □ Yellow bin
□ Red bin □ Other bin
□ Left outside
□ Cannot say

D: INJECTION/BLOOD DRAW

45. Injection: ◻ Intravenous (into vein) ◻ Intramuscular, intradermal or subcutaneous

46. Blood draw: ◻ From vein ◻ Finger or heel prick

E: DISINFECTION/PREPARATION/SEGREGATION

47. Lancet used:
◻ Yes
◻ No

If used:
48. Source:
◻ New (original package)
◻ Reused
◻ Cannot say

49. Disposal
Segregated …
◻ Safety sharps container
◻ Improvised sharps container
◻ Segregated in other bin
◻ Stored for sterilisation
◻ Left outside
◻ Cannot say

50. Needle used:
◻ Syringe ◻ Other
◻ Vacutainer ◻ None
◻ Butterfly

If used:
51. Source:
◻ New (original package)
◻ Reused
◻ Cannot say

52. Disposal:
Segregated …
◻ Safety sharps container
◻ Improvised sharps container
◻ Segregated in other bin
◻ Stored for sterilisation
◻ Left outside
◻ Cannot say

53. Capillary tube used:
◻ Yes
◻ No

If yes:
54. Segregated…
◻ Safety sharps container
◻ Improvised sharps container
◻ Black or blue bin
◻ Yellow bin
◻ Red bin
◻ Other bin
◻ Left outside
◻ Cannot say

55. Glucometer/HB strip used:
◻ Yes
◻ No

If yes:
56. Segregated…
◻ Safety sharps container
◻ Improvised sharps container
◻ Black or blue bin
◻ Yellow bin
◻ Red bin
◻ Other bin
◻ Left outside
◻ Cannot say

57. Work surface:
◻ Clean
◻ Dirty, blood
◻ Dirty, used syringes/needles
◻ Dirty, used swabs/gauze
◻ Dirty, other infectious waste

58. Patient skin prep:
Clean swab…
◻ Spirit dispenser + cotton wool
◻ Spirit bottle + cotton wool
◻ Wet-prep cotton wool
◻ Standardised-alcohol swab
◻ Dirty swab
◻ None

59. Cotton wool (bloodied):
◻ Yes
◻ No

If yes:
60. Segregated…
◻ Safety sharps container
◻ Improvised sharps container
◻ Black or blue bin
◻ Yellow bin
◻ Red bin
◻ Other bin
◻ Left outside or with patient
◻ Cannot say

61. Gloves used:
◻ Yes
◻ No

If yes:
62. Segregated…
◻ Safety sharps container
◻ Improvised sharps container
◻ Black or blue bin
◻ Yellow bin
◻ Red bin
◻ Other bin
◻ Left on hands
◻ Left outside
◻ Cannot say

63. Needle/lancet recapped after use:
◻ No
◻ One hand
◻ Two hands

64. Needle separated:
◻ No
◻ Removal device
◻ Hands

65. HCW had needle/lancet-prick:
◻ No
◻ Yes

F: HAND HYGIENE

66. Are gloves used? Yes ◻ No ◻

If yes:

67. Are gloves…
◻ New ◻ Reused ◻ Cannot say

68. HH directly before gloves:
◻ HR
◻ HW w/soap
◻ HW no soap
◻ None
◻ Cannot say

69. HH directly after gloves:
◻ HR
◻ HW w/soap
◻ HW no soap
◻ None
◻ Cannot say

70. Hand hygiene before procedure:
◻ HR
◻ HW w/soap
◻ HW no soap
◻ None
◻ Cannot say

If yes:

71: Hand hygiene took
_______ seconds

72: Hand hygiene with gloves on:
◻ Yes
◻ No

73: Drying method:
◻ Clean disposable towel
◻ Nothing
◻ Reused towel or clothes
◻ Cannot say

74. Hand hygiene after procedure:
◻ HR
◻ HW w/soap
◻ HW no soap
◻ None
◻ Cannot say

If yes:

75. Hand hygiene took
_______ seconds

76. Hand hygiene with gloves on:
◻ Yes
◻ No

77. Drying method:
◻ Clean disposable towel
◻ Nothing
◻ Reused towel or clothes
◻ Cannot say

78. NOTES/COMMENTS

79. Checked by supervisor (initials)

Procedures – eligibility, consent, data collection

Fieldworkers observed provider-patient interactions for six hours in each facility: three hours in the consultation rooms, one and a half hours in the laboratory, and one and a half hours in the injection or dressing room. All patients were eligible if they or their adult caretaker gave verbal informed consent. Individuals below 18 years of age were excluded if they were not accompanied by an adult caretaker

To minimise bias, fieldworkers were coached to observe discreetly from the corner of the room, limit any interaction with either provider or patient, and not disclose that observations were focused on IPC practices Fieldworkers recorded every indication and action on a paper version of the IPC tool. The data were double entered in Dar es Salaam on tablets using ODK Collect v.1.12.1. Providers were asked for written consent to be observed prior to commencing observation, but were not shown the tool, or told that observation was specifically for the purpose of measuring IPC compliance. PharmAccess staff were informed that IPC compliance would be measured as part of the evaluation, but wwere not given details on how this would be done or access to any of the study tools.

Definition of compliance – domain and indications

Domain and indication	Action for compliance
**Hand hygiene**	
Before touching a patient	Provider washed hands with soap or used alcohol hand rub and did not dry hands on reused towel or clothes.
After touching a patient	
Before a clean/aseptic procedure	
After exposure to body fluids	
Before injection or blood draw	
After injection or blood draw	
**Gloves**	
When using gloves	New gloves were used for each patient
Before putting on gloves	Provider washed hands with soap or used alcohol hand rub
After putting on gloves	Provider washed hands with soap or used alcohol hand rub
When carrying out intravenous injection, blood draw, wound cleaning or dressing	Gloves used
For any other contact with body fluid, mucous membranes or non-intact skin	Gloves used
After using gloves	Gloves discarded into waste bin
**Injection and blood draw safety**	
For each injection or blood draw	New sharp used
For each injection or blood draw	Patient skin prepared with clean swab
For each injection or blood draw	No needle separation, two-handed re-capping or sharps injury

For each injection or blood draw	Work surface was clean of infectious waste
**Disinfection**	
Before or after use of non-infra-red thermometer	Disinfected using rubbing alcohol or bleach
Before or after use of non-disposable tongue depressor	
Before or after use of otoscope	
Before or after use of stethoscope	
**Waste segregation**	
After use of a sharp	Sharp segregated into safety/improvised sharps container
After injection or blood draw which produced infectious waste	Swabs, cotton wool, test strips and capillary tubes disposed into red or yellow waste bin with matching bag, or safety/improvised sharps container
After a medical exam or procedure which produced infectious waste	Swabs, gauze, cotton wool and disposal tongue depressors segregated into red or yellow waste bin with matching bag

SafeCare assessment

The SafeCare score uses data from the SafeCare assessment survey, full details of which can be found at https://www.safe-care.org/who-we-are/safecare-standards/ . Facilties were assessed on 170 (for a basic assessment) or 680 (for an advanced assessment) standards. Each standard was recorded as either non-compliant, partially compliant or fully compliant, aided by a detailed descriptor used to evaluate each practice. We coded “non-compliant” as 0, “partially compliant” as 0.5, and “fully compliant” as 1, and took the unweighted average across the 170 basic standards (applicable in both basic and adavancd assessments) to generate a score.

Health facility survey

Patient volume

Mean monthly patient volume was calculated on the basis of numbers of visits reported for the last three completed months. Fieldworkers were trained to record figures directly from facility records. Data was collected separately for outpatient curative visits, outpatient clinic visits, inpatient admissions (per admission not night stayed) and deliveries. Care which is always free (antenatal visits, postnatal visits, immunisations and child growth checks) was excluded.

Revenue

Mean monthly income was calculated on the basis of revenue reported for the last three completed months. Fieldworkers were trained to record figures directly from facility records. Data was collected separately for cash, mobile money, invoices to insurances companies (on the basis of the last three months for which all invoices had been sent, but payment of the invoices was not required) and government transfers (only cash and not in-kind transfers were included)

Patient exit interviews

Experience of care score

The patient exit survey was used to measure patient satisfaction with the experience of care. We developed a set of 21 questions based on existing tools, including the Hospital Consumer Assessment of HealthCare Providers and Systems done in the US. The questions covered experiences and satisfaction across various dimensions, such as the waiting time, degree of privacy, and ability of the clinician to communicate well. Table B4 provides more details. Fieldworkers read out 21 statements and patients were asked to say whether they agreed, disagreed or neither agreed or disagreed. In accordance with best practice, we used both positively and negatively framed questions to address the upward bias that comes with using all positive questions.

We conducted face-to-face exit interviews with patients as they were about to leave the health facility. A convenience sampling approach was used, with the next available patient approached when a previous interview was finished. Patients were eligible if they had received curative outpatient care (therefore excluding routine visits for growth checks, immunisations or antenatal care) and had completed their visit to the facility (including collecting prescribed treatments and making payments). We sought to interview up to eight patients in each health facility. During interview the fieldworkers entered the data digitally into tablets using ODK Collect v.1.12.1.

To generate a summary score, we first assigned a value of 1 to "agree", 0.5 to "neutral" and 0 to "disagree". For statements that were negatively framed, such as "the clinician did not listen carefully to me", we reverse scored the responses. After normalizing the responses to each question on a 0 to 1 scale, we took the unweighted average across the 21 items. For five of the 21 statements, respondents were permitted to give a "not applicable" response. This response is coded as missing when we take the unweighted average across the items. The summary score is interpreted as the proportion of the maximum satisfaction with the experience of care obtainable.

Table B4: Patient experience questions

	Patient experience statement
1	I had to wait a long time to be served
2	The waiting area was satisfactory
3	The clinician was thorough in investigating my symptoms
4	The clinician did not listen carefully to me
5	The clinician gave me sufficient information about my illness/condition and care
6	The clinician explained things to me in a way that was easy to understand
7	The clinician spent enough time with me
8	It was difficult to find my way to the different rooms I needed to visit in the facility
9	I was attended to in private without being seen or overheard by others
10	I am worried that a patient could pick up an infection from visiting this facility
11	The clinicians who served me seemed highly knowledgeable about my condition
12	Some of the drugs or supplies I was prescribed/ordered were not available at the facility
13	All the services I needed were available at this facility

14	The facility appears well managed and organised
15	I was given clear information on how to take the medicines I've received
16	I understood the fees I was charged
17	I trust the staff here to act in my best interests
18	The facility is run down or in a poor state of repair
19	The facility is clean
20	The staff at the facility, including at reception and the pharmacy, were polite to me
21	The services were reasonably priced

Appendix C: SafeCare programme

Components

QIP progress visits

A key part of the SafeCare intervention is the mentoring visits made by the quality advisors to health facilities. They are described as quality improvement plan (QIP) progress visits, as the main aim is for the advisor to monitor the facility's progress towards implementing its QIP. The target frequency for visits is approximately once per quarter, which can be translated into at least five visits during the study period for each intervention facility. The distribution of number of visits received by implementing partner is shown in Figure C1. The mean number of visits received was 3.1 overall, 4.0 for APHFTA facilities and 2.2 for CSSC facilities ($p<0.0001$).

Figure C1: Mentoring visits in intervention facilities, by implementation partner

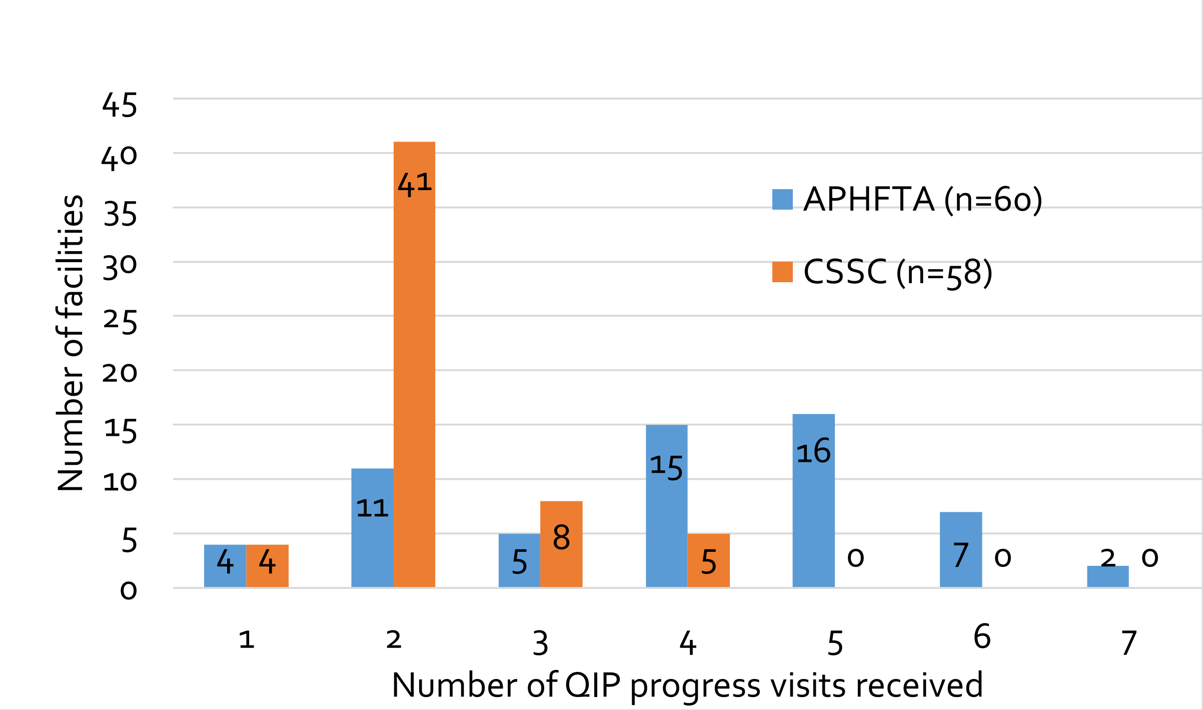

Training sessions

Training sessions could be delivered at external venues attended by multiple facilities, or on-site at a given facility, though informal training during mentoring visits was generally not recorded. It was expected that each facility receives at least two trainings during the intervention, one on quality and one on business. The mean number of trainings attended was 0.6 overall, 1.1 for APHFTA facilities and 0.1 for CSSC facilities. Details are given in Table C1 below.

Table C1: Training sessions in intervention facilities by implementation partner

TRAININGS ATTENDED	APHFTA (N=60)	CSSC (N=58)	TOTAL (N=118)
**0**	14	52	66
**1**	31	5	36
**2**	13	1	14
**3**	2	0	2

Loans

Loans, provided by commercial banks but guaranteed by the Medical Credit Fund (MCF), were part of the intervention package but in practice available to both control and intervention facilities. The details of the four loans disbursed in the study period (two to intervention facilities, two to control facilities) are given below.

Table C2: Details of loans

Study Arm	*Implementation partner*	*Month of disbursement*	*Loan size*
Control	APHFTA	April 2018	10m TSH<br>US $4,264
Control	APHFTA	April 2018	20m TSH<br>US $8,529
Intervention	APHFTA	April 2018	150m TSH<br>US $63,966
Intervention	APHFTA	October 2017	40m TSH<br>US $17,058

Theory of change

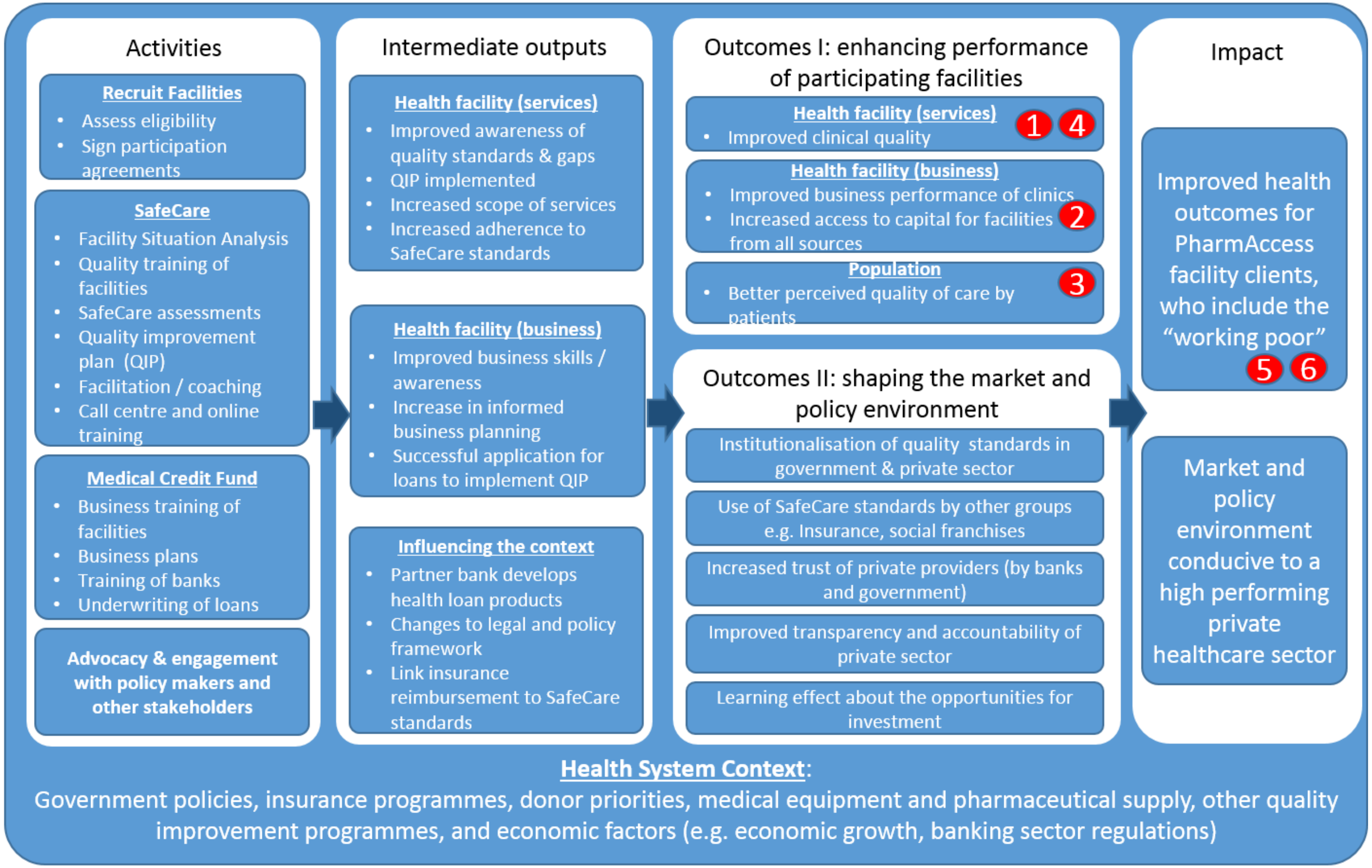

Key Assumptions:

1. Health providers are motivated to improve quality either because they are intrinsically motivated or they perceive there to be financial rewards.
2. Health providers' lack of business and managerial skills and poor access to credit are constraints on the development of their facility.
3. Quality of care changes are perceived by patients.
4. Improvement in SafeCare standards leads to improved clinical quality.
5. Improvements in technical process quality take place in areas with substantial impact on patient morbidity and mortality.
6. Health providers in PharmAccess programs serve the working poor.

Appendix D: Additional results

Table D1. Attrition: baseline balance in each sample surveyed

Variable	Endline SafeCare assessment facilities (n=221)		Standardised patient facilities (n=227)		IPC observation facilities (n=220)	
	Intervention	Control	Intervention	Control	Intervention	Control
Number of facilities	109	112	111	116	106	114
Baseline SafeCare assessment score, % of max (SD)	42.4 (12.6)	42.1 (12.0)	42.3 (12.6)	41.8 (11.8)	42.9 (12.3)	42.1 (12.1)
Partner organisation						
APHFTA	52 (47.7%)	57 (52.3%)	54 (48.7%)	56 (48.3%)	52 (49.1%)	57 (50.0%)
CSSC	53 (47.3%)	59 (52.7%)	57 (51.4%)	60 (51.7%)	54 (50.9%)	57 (50.0%)
Facility level						
Dispensary	59 (54.1%)	62 (55.4%)	60 (54.1%)	65 (56.0%)	55 (51.9%)	64 (56.1%)
Health Centre	33 (31.1%)	32 (28.1%)	33 (29.7%)	33 (28.5%)	33 (31.1%)	32 (28.1%)
Hospital	18 (16.5%)	18 (16.1%)	18 (16.2%)	18 (15.5%)	18 (17.0%)	18 (15.8%)
Facility location						
Inside Dar Es Salaam	19 (17.4%)	19 (17.0%)	21 (18.9%)	21 (18.1%)	21 (19.8%)	20 (17.5%)
Outside Dar Es Salaam	90 (82.6%)	93 (3.0%)	90 (81.1%)	95 (81.9%)	85 (80.2%)	94 (82.5%)
Location type						
Urban	35 (32.1%)	35 (32.3%)	38 (34.3%)	32 (27.6%)	35 (33.0%)	34 (29.8%)
Peri-urban	28 (25.7%)	30 (26.8%)	28 (25.2%)	33 (28.5%)	27 (25.5%)	33 (29.0%)
Rural	46 (42.2%)	47 (42.0%)	45 (40.5%)	51 (44.0%)	44 (41.5%)	47 (41.2%)
Staffing and infrastructure						
Number of medical doctors	1.3 (2.1)	1.2 (1.8)	1.3 (2.2)	1.2 (1.8)	1.4 (2.2)	1.3 (1.8)
Number of clinical officers	2.2 (2.3)	1.9 (1.6)	2.2 (2.3)	1.9 (1.6)	2.2 (2.3)	1.9 (1.6)
Number of nurses and midwives	7.1 (13.1)	6.3 (11.0)	7.1 (13.0)	6.2 (10.9)	7.3 (13.3)	6.2 (11.0)
Number of total staff	30.7 (40.5)	28.2 (37.6)	30.5 (40.2)	27.7 (37.1)	31.6 (40.8)	28.1 (37.3)
Number of consulting rooms	2.4 (2.6)	2.4 (1.9)	2.4 (2.6)	2.3 (1.8)	2.4 (2.6)	2.4 (1.8)
Number of inpatient admission beds	32.9 (57.7)	25.3 (48.4)	32.4 (57.3)	24.8 (47.8)	33.9 (58.2)	24.9 (48.2)
Open 24 hours, 7 days a week	66 (62.3%)	73 (64.0%)	69 (62.2%)	75 (64.7%)	66 (62.3%)	73 (64.0%)

Data are mean (SD) or n (%). APHFTA is the Association of Private Health Facilities in Tanzania. CSSC is the Christian Social Services Commission. Data collected by PharmAccess as part of intervention activities, in SafeCare Assessment or Situational Analysis.

Table D2. Effect on endline SafeCare assessment score by domain and service element

Outcomes	Intervention	Control	Absolute difference (95% CI)	P value
Endline SafeCare assessment score	55.2%	50.8%	4.4% (0.9 to 7.7)	0.015
By service element				
Governance & Management	51.6%	48.0%	3.3% (-0.8 to 7.3)	0.111
Human Resource Management	35.1%	27.8%	7.4% (2.0 to 12.8)	0.008
Patient and Family Rights & Access to Care	66.5%	59.6%	6.9% (2.9 to 10.8)	0.001
Management of Information	66.2%	63.2%	2.3% (-2.0 to 6.7)	0.294
Risk Management	35.8%	29.1%	6.9% (2.5 to 11.2)	0.002
Primary Healthcare (Outpatient) Services	62.9%	60.0%	3.0% (-1.3 to 7.2)	0.170
In-patient Care	51.5%	41.0%	9.1% (2.4 to 15.8)	0.008
Surgery & Anesthesia Services	58.3%	54.6%	4.8% (-6.0 to 15.7)	0.376
Laboratory Services	67.0%	62.6%	4.9% (-0.2 to 10.1)	0.061
Diagnostic Imaging Services	78.2%	75.4%	3.0% (-8.7 to 14.8)	0.608
Medication Management	54.8%	52.6%	1.8% (-2.2 to 5.9)	0.371
Facility Management Services	57.0%	54.6%	1.9% (-2.2 to 6.1)	0.363
Support Services	32.1%	26.6%	5.5% (0.8 to 10.2)	0.023

Table D3. Number of IPC indications by study arm

Variable	Intervention	Control	P value
Mean (96% CI) number of IPC indications per patient observation	5.51 (5.33 – 5.69)	5.41 (5.24 – 5.58)	0.426
Hand hygiene indications	1.61 (1.56 – 1.66)	1.58 (1.53 – 1.63)	0.372
Glove use indications	1.29 (1.24 – 1.34)	1.26 (1.21 – 1.31)	0.326
Injection and blood draw indications	1.62 (1.55 – 1.70)	1.60 (1.53 – 1.67)	0.714
Disinfection indications	0.16 (0.14 – 0.18)	0.15 (0.14 – 0.17)	0.470
Waste segregation indications	0.83 (0.79 – 0.86)	0.82 (0.78 – 0.85)	0.806

P values for differences in mean indications per patient are from a univariate linear regssion model

Table D4. Characteristics of patients observed for IPC

Variable	Intervention	Control
Number of patients observed	1713	1804
Age		
<5	329 (19.2%)	344 (19.1%)
5-17	217 (12.7%)	232 (12.9%)
18-29	414 (24.2%)	419 (23.2%)
30-49	413 (24.1%)	457 (25.3%)
50+	248 (14.5%)	245 (13.6%)
Unknown	92 (5.4%)	107 (5.9%)
Sex		
Male	742 (43.3%)	754 (41.8%)
Female	971 (56.7%)	1050 (58.2%)

Table D5. Effect on IPC compliance adjusted for patient mix and indication

Model	Intervention	Control	Absolute difference (95% CI)	Odds ratio (95% CI)	P value
Baseline (model 1)	8260 / 16289 (50.7%)	8448 / 17237 (49.0%)	2.1% (0.1% - 4.0%)	1.09 (1.00 – 1.18)	0.044
Adjusted for patient mix (model 2)	8260 / 16289 (50.7%)	8448 / 17237 (49.0%)	2.2% (0.2% - 4.2%)	1.09 (1.00 – 1.18)	0.035
Adjusted for patient mix and indications (model 3)	8260 / 16289 (50.7%)	8448 / 17237 (49.0%)	1.9% (-0.1% - 4.0%)	1.08 (1.00 - 1.17)	0.065

Data are n / N (%), unless specified. The absolute difference is the marginal effect in percentage points from a multilevel mixed effects logistic regression that included, in the baseline model, facility random effects to take into account the clustered randomisation design and stratum fixed effects. Model 2 adjusts for patient age and patient sex. Model 3 adjusts for patient age, patient sex, and indication. The odds ratios are from the corresponding multilevel mixed effects logistic regressions.

Hawthorne effect sensitivity analysis

Directly observing health workers gives rise to the possibility that they alter their behaviour. The specific concern in our study is that health workers may have temporarily increased IPC compliance when they were observed by the research team, above their usual level of performance. We assessed the presence of a Hawthorne effect by examining whether compliance with IPC practices was associated with order number of patients observed. The general idea behind the sensitivity analysis is that, if there was a Hawthorne effect, health workers would eventually get used to being observed and IPC compliance would revert to a lower level closer to actual practice. In the data this would show up as a negative association between IPC compliance and order of patient observation. While evidence of a Hawthorne effect has implications for measurement, it is only a concern for the integrity of the trial if the association between compliance with IPC practices and the order number of patients observed differs by treatment arm.

We analysed data at the level of patient observation. The dependent variable was IPC compliance, defined as the proportion of indications for which the health worker complied with the correct action. The independent variable of interest was the order number of patients observed for each health worker. Since there were observations with an order number of patients observed above 15, we recoded these as “15 or more” in the descriptive figures. Each health worker was observed for an average of 5.0 patient interactions (SD 4.3).

To test for the presence of a Hawthorne effect, we ran four OLS regression models. Model 1 included no other covariates. Model 2 included an indicator for each IPC indication. Model 3 additionally controlled for health worker (age, sex, cadre) and facility (level, ownership, location) characteristics. Model 4 replaced the facility and health worker characteristics with health worker fixed effects, and is our preferred model. To test for whether any association between compliance with IPC practices and the order number of patients observed differs by treatment arm, we ran equivalent OLS regressions, additionally including the treatment dummy, and an interaction between the treatment dummy and the order number of patients observed. The coefficient on the interaction is the coefficient of interest.

Figure D1 shows IPC compliance by order of patient observation in the treatment and control groups separately. Two results are worth noting. First, there is an increasing trend: IPC compliance tends to be better at higher order number of patients observed but the trend is clearly very modest. It is important to note that Figure D1 presents descriptive data and the relationship is likely confounded by the fact that more patients (per health worker) were observed in the laboratory where IPC compliance was consistently high by the very nature of the IPC indications triggered when drawing blood. Second, the relationship between IPC compliance and order number of patients observed is very similar between treatment and control.

Table D6 reports the first set of regression results. Across the four models, there is no consistent direction in the association and the coefficient is small in magnitude in all instances. In the unadjusted model, the coefficient is positive (p=0.011), mirroring the pattern shown in Figure D1. In model 4 – our preferred model with indicators for each indications and health worker fixed effects – the coefficient is negative and insignificant (p=0.862). Table D7 includes an interaction to test whether the linear trend differs between treatment and control. In the unadjusted model, the trend in the treatment group is not significantly different from that of the control group (p=0.627). This result is confirmed when we include health worker fixed effects in model 4 (p=0.182).

Figure D1. IPC compliance and order of patient observation by treatment group

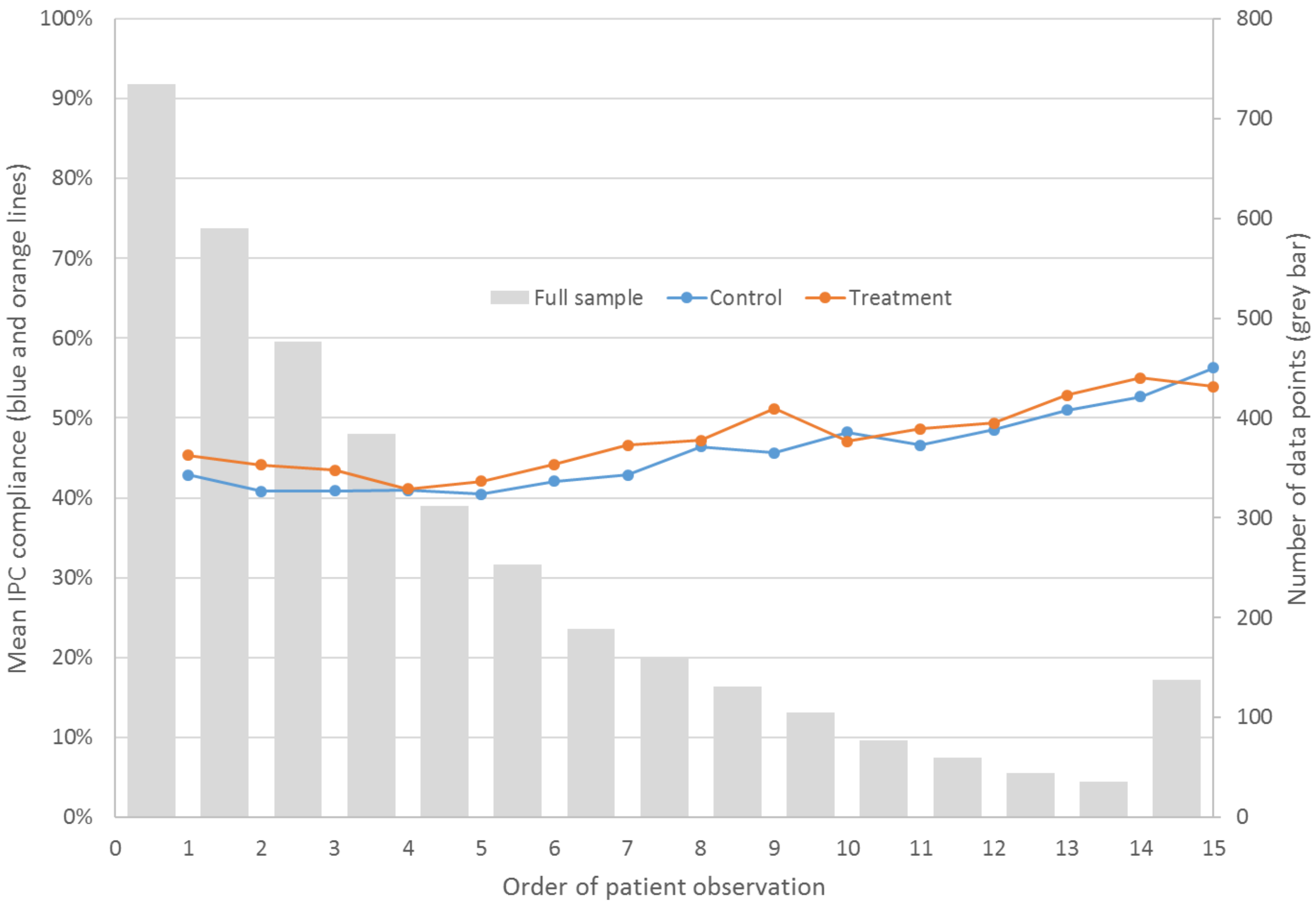

Table D6. Testing for a Hawthorne effect: is compliance associated with order number of patients observed?

	Model 1: unadjusted		Model 2: IPC indication		Model 3: facility and health worker characteristics		Model 4: health worker fixed effects	
	Coefficient (95%CI)	**P value**	**Coefficient (95%CI)**	**P value**	**Coefficient (95%CI)**	**P value**	**Coefficient (95%CI)**	**P value**
Order number of patients observed	0.007 (-0.002 to 0.012)	0.011	-0.004 (-0.006 to -0.002)	<0.001	-0.002 (-0.004 to -0.0001)	0.036	-0.0001 (-0.001 to 0.001)	0.862
Number of observations	3688		3688		3688		3688	
Number of facilities	220		220		220		220	

Table D7. Testing for a Hawthorne effect by treatment arm

	Model 1: unadjusted		Model 2: IPC indication		Model 3: facility and health worker characteristics		Model 4: health worker fixed effects	
	**Coefficient (95%CI)**	**P value**	**Coefficient (95%CI)**	**P value**	**Coefficient (95%CI)**	**P value**	**Coefficient (95%CI)**	**P value**
Order number of patients observed	0.008 (0.002 to 0.014)	0.006	-0.004 (-0.007 to -0.002)	0.001	-0.003 (-0.005 to 0.00003)	0.053	0.0007 (-0.001 to 0.002)	0.408
Treatment	0.032 (-0.017 to 0.082)	0.203	0.015 (-0.007 to 0.038)	0.186	0.016 (-0.006 to 0.038)	0.153	(omitted)	
Order number X treatment	-0.003 (-0.013 to 0.008)	0.627	-0.00003 (-0.004 to 0.004)	0.988	0.001 (-0.003 to 0.005)	0.641	-0.002 (-0.004 to 0.0008)	0.182
Number of observations	3688		3688		3688		3688	
Number of facilities	220		220		220		220	

Notes: Table shows results from OLS regressions in which standard errors are clustered at the level of facility. Data are analysed at the level of patient observation. The dependent variable is IPC compliance defined as the proportion of indications for which the health worker complied with the correct action. The independent variable of interest is the order number of patients observed. Each health worker was observed for an average of 4.9 patient interactions (SD 4.2). Model 1 includes no other covariates. Model 2 includes an indicator for each IPC indication. Model 3 additionally controls for facility and health worker characteristics. Model 4 replaces the facility and health worker characteristics with health worker fixed effects.

SP detection survey

Detais of the detection survey are given in the SP protocol in Appendix B. Interviews were completed with 225 facilities representing 901 visits. A visit was coded as a confirmed detection if the facility reported receiving an SP visit and gave the name used by the SP. Possible detection was coded if the facility gave details which matched the visit (symptoms, gender, approximate age or date) but not the name. Visits were categorised as not detected if the facility did not report any suspicion of having an SP visit, or reported suspicions which did not match the details of the actual SP visits. Results of the detection survey are given in Table D8. SP results excluding detected SPs are given in Table D9.

Table D8. SP detection survey results

		Number of SP visits	No detection	Possible detection	Confirmed detection
All facilities		901	853 (94.7%)	9 (1.0%)	39 (4.3%)
By study arm	Treatment	440	414 (94.1%)	3 (0.7%)	23 (5.2%)
	Control	461	439 (95.2%)	6 (1.3%)	16 (3.4%)

Table D9: Effect on SPs excluding confirmed and possible detections

Outcomes	Intervention	Control	Absolute difference (95% CI)	Odds ratio (95% CI)	P value
Overall correct case management	112/ 418 (26.8%)	128 / 443 (28.9%)	-2.4% (-8.4 – 3.6)	0.88 (0.65 – 1.20)	0.429
Asthma correct management	7 / 104 (6.7%)	6 / 109 (5.5%)	1.3% (-5.8 – 8.3)	1.21 (0.42 – 3.50)	0.725
Non-malarial febrile illness correct management	75 / 104 (72.1%)	80 / 112 (71.4%)	0.9% (-11.1 – 13.0)	1.05 (0.57 – 1.93)	0.885
Tuberculosis correct management	21 / 105 (20.0%)	30 / 112 (26.8%)	-7.8% (-18.9 – 3.4)	0.64 (0.33 – 1.22)	0.176
Upper respiratory tract infection correct management	9 / 105 (8.6%)	12 / 110 (10.9%)	-2.8% (-11.2 – 5.7)	0.75 (0.31 – 1.82)	0.523

Sub-group analyses

Table D10: SP case management (proportion of SPs who received correct management)

Model	Intervention	Control	Absolute difference (95% CI)	Odds ratio (95% CI)	P value
APHFTA	28/216 (22.2%)	55/225 (24.4%)	-2.3 (-10.3 to 5.6)	0.88 (0.56 to 1.37)	0.567
CSSC	72/228 (31.6%)	81/240 (33.8%)	-3.2 (11.7 to 5.4)	0.86 (0.58 to 1.28)	0.467
Dispensary	57/240 (23.8%)	60/260 (23.1%)	0.0 (-7.4 to 7.4)	1.00 (0.66 to 1.52)	0.999
Health Centre	36/132 (27.3%)	47/133 (35.3%)	-8.2 (-19.6 to 3.2)	0.68 (0.39 to 1.17)	0.163
Hospital	27/72 (37.5%)	29/72 (40.3%)	-2.2 (-18.3 to 13.8)	0.91 (0.46 to 1.80)	0.785
Low Safecare assessment baseline score	48/208 (23.1%)	60/240 (25.0%)	-2.8 (-10.8 to 5.1)	0.85 (0.55 to 1.33)	0.485
High Safecare assessment baseline score	72/236 (30.5%)	76/225 (33.8%)	-3.3 (-12.0 to 5.4)	0.86 (0.57 to 1.29)	0.456
Low competition	65 / 224 (29.0%)	77 / 240 (32.1%)	-3.1 (-11.7 to 5.5)	0.862 (0.57 to 1.30)	0.483
High competition	55 / 220 (25.0%)	59 / 225 (26.2%)	0.3 (-8.4 to 9.0)	1.015 (0.63 to 1.63)	0.951

Table D11: IPC compliance (propotion of IPC indications with corresponding compliant action)

Model	Intervention	Control	Absolute difference (95% CI)	Odds ratio (95% CI)	P value
APHFTA	3662/6335 (57.8%)	4000/7057 (56.7%)	1.7 (-2.1 to 5.4)	1.07 (0.92 to 1.25)	0.390
CSSC	4551/8031 (56.7%)	4397/8185 (53.7%)	3.2 (-0.1 to 6.4)	1.14 (1.00 to 1.30)	0.055
Dispensary	3527/6070 (58.1%)	4053/7165 (56.6%)	2.0 (-1.6 to 5.6)	1.09 (0.94 to 1.26)	0.273

Health Centre	2784/4819 (57.8%)	2350/4453 (52.8%)	6.5 (2.8 to 10.3)	1.31 (1.12 to 1.53)	0.001
Hospital	1902/3477 (54.7%)	1994/3624 (55.0%)	0.2 (-4.4 to 4.9)	1.01 (0.84 to 1.22)	0.926
Low Safecare assessment baseline score	3193/5538 (57.7%)	3524/6569 (52.5%)	5.1 (1.4 to 8.8)	1.23 (1.06 to 1.44)	0.007
High Safecare assessment baseline score	5020/8828 (56.9%)	4883/8673 (56.3%)	0.4 (-2.9 to 3.8)	1.01 (0.89 to 1.16)	0.829
Low competition	3964 / 7871 (50.4%)	4758 / 9839 (48.4%)	1.4 (-1.5 to 4.4)	1.06 (0.94 to 1.19)	0.343
High competition	4296 / 8418 (51.0%)	3690 / 7398 (49.9%)	1.1 (-1.8 to 3.9)	1.04 (0.93 to 1.17)	0.468

Additional quality support

Table D12: Quality improvement support received from sources other the SafeCare reported at endline

	All facilities (n=228)	Intervention (n=111)	Control (n=117)
Had any visit from Government's Star Rating programme in last two years	214 (93.9%)	104 (93.7%)	110 (94.0%)
Mean visits received from Government's Star Rating in last two years	1.67	1.57	1.77
Had any other visit from Minisitry of Health in last two years	203 (89.0%)	98 (88.3%)	105 (89.7%)
Current participant of other quality improvement programme[1]	166 (72.8%)	81 (73.0%)	85 (72.7%)
Mean number of current other quality improvement programmes	1.26	1.29	1.23

[1]Facilities were asked whether they were part the following programmes, or any other programme to improve quality or increase scope of services: Familia/Population Services International, Blue Star/Marie Stopes International, Trust/DKT, Kaizen/5S, Afya Microfinance, and Results Based Financing.

Robustness checks for business outcomes

Table D13: Utilisation and revenue outcomes using logged values

Outcomes	Difference (95% CI)	P value
Facility patient volume, visits per month (natural log)	0.04 (-0.24 to 0.32)	0.789
Outpatient visits (natural log)	-0.06 (-0.22 to 0.34)	0.670
Inpatient admissions (natural log)	-0.01 (-0.38 to 0.37)	0.964
Facility revenue, USD per month (natural log)	0.08 (-0.24 to 0.41)	0.610
Cash user fee revenue (natural log)	0.03 (-0.39 to 0.44)	0.897
Insurance revenue (natural log)	0.07 (-0.84 to 0.98)	0.877

Other revenue sources (natural log)	-0.16 (-0.75 to 0.43)	0.597

Estimates and p values are from a linear regression model with robust standard errors, controlling for study strata

Table D14: Utilisation and revenue outcomes trimmed at the 98th percentile

Outcomes	Difference (95% CI)	P value
Facility patient volume, visits per month	32 (-172 to 236)	0.755
Outpatient visits	46 (-145 to 237)	0.635
Inpatient admissions	-4 (-27 to 19)	0.726
Facility revenue, USD per month	-570 (-2362 to 1223)	0.532
Cash user fee revenue	-215 (-1226 to 796)	0.676
Insurance revenue	265 (-617 to 1046)	0.505
Other revenue sources	99 (-118 to 316)	0.369

Estimates and p values are from a linear regression model with robust standard errors, controlling for study strata

Table D15: Utilisation and revenue outcomes trimmed at the 95th percentile

Outcomes	Difference (95% CI)	P value
Facility patient volume, visits per month	-15 (-179 to 150)	0.861
Outpatient visits	18 (-136 to 171)	0.821
Inpatient admissions	-9 (-25 to 7)	0.270

Facility revenue, USD per month	320 (-1144 to 1784)	0.667
Cash user fee revenue	277 (-544 to 1099)	0.506
Insurance revenue	58 (-532 to 648)	0.847
Other revenue sources	-40 (-152 to 72)	0.480

Estimates and p values are from a linear regression model with robust standard errors, controlling for study strata

Table D15: Utilisation and revenue outcomes using the three montly data points for each as separate observations

Outcomes	Difference (95% CI)	P value
Facility patient volume, visits per month	119 (-123 to 362)	0.333
Outpatient visits	150 (-79 to 380)	0.198
Inpatient admissions	-2 (-56 to 58)	0.954
Facility revenue, USD per month	1592 (-1618 to 4802)	0.329
Cash user fee revenue	1001 (-1729 to 3731)	0.471
Insurance revenue	630 (-706 to 1966)	0.354
Other revenue sources	217 (-381 to 814)	0.475

Data was reshaped wide to long to give three values per facility for each outcome. Estimates and p values are from a linear regression model controlling for study strata, clustered at the facility to account for the multiple observations per facility.
